# Multilocus loss of DNA methylation in individuals with mutations in the histone H3 Lysine 4 Demethylase KDM5C

**DOI:** 10.1186/1755-8794-6-1

**Published:** 2013-01-28

**Authors:** Daria Grafodatskaya, Barian HY Chung, Darci T Butcher, Andrei L Turinsky, Sarah J Goodman, Sana Choufani, Yi-An Chen, Youliang Lou, Chunhua Zhao, Rageen Rajendram, Fatima E Abidi, Cindy Skinner, James Stavropoulos, Carolyn A Bondy, Jill Hamilton, Shoshana Wodak, Stephen W Scherer, Charles E Schwartz, Rosanna Weksberg

**Affiliations:** 1 Genetics and Genome Biology Program, Hospital for Sick Children, Toronto, ON, Canada; 2 Division of Clinical and Metabolic Genetics, Hospital for Sick Children, Toronto, ON, Canada; 3 Centre of Reproduction, Growth & Development, Department of Pediatrics & Adolescent Medicine, The University of Hong Kong, Hong Kong, Hong Kong; 4 Program in Molecular Structure and Function, Hospital for Sick Children, Toronto, ON, Canada; 5 J.C. Self Research Institute, Greenwood Genetic Center, Greenwood, SC, USA; 6 Department of Pediatric Laboratory Medicine, Hospital for Sick Children, Toronto, ON, Canada; 7 Developmental Endocrinology Branch, National Institute of Child Health and Human Development, National Institutes of Health, Bethesda, MD, USA; 8 Division of Endocrinology, Department of Pediatrics, Hospital for Sick Children, Toronto, ON, Canada; 9 Department of Pediatrics, University of Toronto, Toronto, ON, Canada; 10 Department of Molecular and Medical Genetics, University of Toronto, Toronto, ON, Canada; 11 The Centre for Applied Genomics, Hospital for Sick Children, Toronto, ON, Canada

**Keywords:** *KDM5C*, DNA methylation, H3K4 methylation, Intellectual disability

## Abstract

**Background:**

A number of neurodevelopmental syndromes are caused by mutations in genes encoding proteins that normally function in epigenetic regulation. Identification of epigenetic alterations occurring in these disorders could shed light on molecular pathways relevant to neurodevelopment.

**Results:**

Using a genome-wide approach, we identified genes with significant loss of DNA methylation in blood of males with intellectual disability and mutations in the X-linked *KDM5C* gene, encoding a histone H3 lysine 4 demethylase, in comparison to age/sex matched controls. Loss of DNA methylation in such individuals is consistent with known interactions between DNA methylation and H3 lysine 4 methylation. Further, loss of DNA methylation at the promoters of the three top candidate genes *FBXL5*, *SCMH1*, *CACYBP* was not observed in more than 900 population controls. We also found that DNA methylation at these three genes in blood correlated with dosage of *KDM5C* and its Y-linked homologue *KDM5D*. In addition, parallel sex-specific DNA methylation profiles in brain samples from control males and females were observed at *FBXL5* and *CACYBP*.

**Conclusions:**

We have, for the first time, identified epigenetic alterations in patient samples carrying a mutation in a gene involved in the regulation of histone modifications. These data support the concept that DNA methylation and H3 lysine 4 methylation are functionally interdependent. The data provide new insights into the molecular pathogenesis of intellectual disability. Further, our data suggest that some DNA methylation marks identified in blood can serve as biomarkers of epigenetic status in the brain.

## Background

A number of neurodevelopmental syndromes are caused by mutations in genes encoding proteins involved in epigenetic regulation [1,2]. Loss of function of proteins encoded by such genes is expected to result in alterations of epigenetic marks at specific genomic loci. To test this hypothesis, we elected to study the X-linked gene *KDM5C,* encoding histone H3 lysine 4 (H3K4) demethylase*.* Mutations in the *KDM5C* gene (MIM No: 314690) were first described as causing X-linked intellectual disability (XLID) in 2005 [3]. To date, 21 different *KDM5C* mutations have been identified in XLID patients. The prevalence of *KDM5C* mutations in patients with XLID is estimated to be ~3% [3-10]. The clinical features most consistently reported in males with mutations include mild to severe intellectual disability (ID), epilepsy, short stature, hyperreflexia, aggressive behavior and microcephaly. In addition, a mutation in *KDM5C* was identified in one male case of autism [11]. Female mutation carriers are usually unaffected but sometimes demonstrate mild ID or learning difficulties [7].

**Figure 1:**
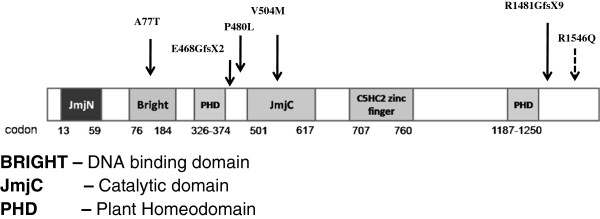
**Schematic diagram of the human KDM5C protein.** The diagram shows the functional domains and the positions of 5 mutations as well as the p.R1546Q variant of unknown clinical significance.

KDM5C is a member of the evolutionarily conserved KDM5 family of four proteins, KDM5A/B/C and D. KDM5A/C/D demethylate tri- and di-methylated forms of H3K4, whereas KDM5B is capable of demethylating all three forms (tri-, di-, and mono) of H3K4 methylation [12,13]. The KDM5C protein contains several conserved functional domains, including the Bright/ARID DNA binding domain; the catalytic JmjC domain; the JmjN domain responsible for protein stability; the zinc finger-C5HC2 domain; and two PHD domains, responsible for histone binding [14] (Figure 1). Mutations leading to XLID have been found in most of the functional domains of this protein [15]*. KDM5C* is ubiquitously expressed in almost all human tissues including white blood cells, with the highest levels of expression found in the brain and in skeletal muscle [3,15].

A significant effort has been invested in elucidating the role of *KDM5C* mutations in the ID phenotype. In zebrafish, downregulation of *KDM5C* leads to an increase in neuronal cell death and a decrease in total dendritic length [13]. Chromatin immune precipitation (ChIP) of HeLa cells revealed that KDM5C co-localizes with a transcriptional repressor REST, in the promoters of a subset of REST target genes, suggesting that loss of KDM5C activity impairs REST-mediated neuronal gene regulation [16]. ChIP-sequencing of a panel of chromatin remodeling proteins in the leukemia cell line K562 had shown that KDM5C along with other transcriptional repressors binds to a wide range of promoters, including those that are active, competent, and repressed [17]. The specific molecular mechanism by which loss of function of *KDM5C* causes impairment in neuronal development is not understood, but epigenetic deregulation is presumed to play an important role.

The KDM5C protein is likely to play a role not only in ID but also in sex-specific differences in brain function. The X-linked human *KDM5C* and its mouse ortholog *Kdm5c* escape X–inactivation [18,19]; and, not surprisingly, *Kdm5c* has higher expression levels in XX females compared to XY males in mouse adult brain [20]. This difference has been shown to be associated with sex chromosome complement (XX vs. XY), rather than gonadal sex of the animals [21]. Interestingly, there is a Y-linked functional homologue of *KDM5C*, namely *KDM5D*, in both human and mouse. The homologue in murine neurons, *Kdm5d* has been shown to be expressed at lower levels than *Kdm5c* and is not able to compensate for *Kdm5c* differences between females and males [21].

Recent studies suggest there is interplay between histone modifications and DNA methylation [22,23]. This relationship is bidirectional; histone modifications are more labile while DNA methylation is more stable [22]. In embryonic development, the formation of histone marks precedes and guides *de novo* DNA methylation, either by recruiting *de novo* DNA methyltransferase enzymes (H3K9 methylation) [24], or by protecting DNA from *de novo* methylation (H3K4 methylation) [25,26].

We hypothesized that in patients with *KDM5C* mutations an aberrant increase of H3K4 tri- and di-methylation leads to decreased DNA methylation at genomic sites critical for normal neurodevelopment. We also proposed that the sites exhibiting decreased DNA methylation due to *KDM5C* mutation would also exhibit sexually dimorphic patterns of DNA methylation correlating with *KDM5C* and *KDM5D* dosage in normal females and males. Since *KDM5C* escapes X-inactivation [18], we enriched our sample set by including 47,XXX, 47,XXY and 45,X (Turner syndrome) individuals. In agreement with our hypothesis, we identified a significant loss of DNA methylation at specific genomic loci in blood samples of male patients carrying *KDM5C* mutations, suggesting these genes are epigenetic targets of KDM5C. To our knowledge, this is the first report of significant DNA methylation alterations in association with a mutation in a human histone modifying enzyme. Furthermore, we have shown that some genes with downstream loss of DNA methylation in individuals with *KDM5C* mutation also demonstrate positive correlation for DNA methylation levels across individuals with varying *KDM5C/KDM5D* dosage due to sex chromosome number in both blood and brain.

## Methods

### Research subjects

This study was approved by research ethics boards of the Greenwood Genetic Center (Greenwood, SC, USA) and the Hospital for Sick Children (Toronto, ON, Canada). All research subjects and/or their caregivers provided informed consent. Blood samples were collected from 10 patients with XLID and confirmed *KDM5C* mutations [5], two patients with XLID and *KDM5C* sequence variant, 19 unaffected control males (three unaffected relatives/16 unrelated individuals) (Additional file 1: Table S1), 13 control females, 11 females with Turner syndrome (45,X karyotype), three males with 47,XXY karyotype and three females with 47,XXX karyotype. DNA was extracted from 5 ml of blood using phenol-chloroform and ethanol precipitation.

### Methylation microarray

The HumanMethylation27 BeadChip (Illumina, San Diego, CA) containing 27,578 individual CpG sites covering >14,000 genes was used for genome-wide DNA methylation analysis. Genomic DNA was sodium bisulfite converted with the EpiTect Bisulfite Kit according to the manufacturer’s protocol (Qiagen, Germantown, MD). Labeling, hybridization and scanning were performed at the Centre for Applied Genomics (TCAG) at The Hospital for Sick Children, Toronto, Canada. The methylation status of the interrogated CpG sites was measured from the intensity values of the methylated (M) and unmethylated (U) probes, as the ratio of fluorescent signals β = *Max*(*M*,0)/*Max*(*M*,0) + *Max*(*U*,0) + 100]. DNA methylation β values are continuous variables between 0 (absent methylation) and 1 (completely methylated) representing the ratio of combined locus intensity. The β values were extracted using the Methylation Module in Illumina Bead Studio after background normalization. All samples included in the analysis passed quality control metrics including bisulfite conversion control intensity values in green channel >4000 and 99% of probes had p-values of detection of signal above background <0.01 [27,28]. 6 publically available datasets (accession numbers: GSE19711, GSE36064, GSE27097, GSE25395, GSE20067, GSE20236) were used to assess DNA methylation at *FBXL5* (cg02630888), *SCMH1* (cg03387723), and *CACYBP* (cg16743289) CpG sites. Sex specific DNA methylation analysis in brain was performed using published dataset (GEO Accession No: GSE15745) [29]. If the QC information was available, only samples with appropriate QC metrics (BS control intensity values in green channel >4000 and ≥95% of probes with p-values of detection of signal above background <0.05) were included in the analysis.

### Statistical analysis

Microarray probes with detection p-value ≥ 0.01 [28], 2,984 cross-reactive probes, and 907 probes overlapping SNPs in queried CpG [30] were excluded, leaving in total 23,837 sites for downstream statistical analysis. A non-parametric Mann–Whitney *U* test was used for group comparisons. To adjust for multiple comparisons, a permutation-based method controlling the false discovery proportion (FDP) γ in the data, for a pre-specified confidence levels α was applied [31]. 1000 random permutations of the mutation labels among the data cases, while maintaining the sample sizes (10 vs. 19) were generated. For each permutation Mann–Whitney *U* test-based p-values for all CpG sites were ranked from smallest to largest (from most significant to least significant). The distribution of the 1000 “best” permutation-based p-values was used to determine p-values cut offs for different confidence levels based on the percentiles of this distribution. The cut off p-values were used to determine the number of significant CpG sites at different γ and α level. The Principal Component Analysis was performed in R statistical package using median centering of the data as well as with missing-value imputation to the 10 nearest neighbors.

### Bisulfite pyrosequencing

Targeted DNA methylation analysis was performed using pyrosequencing as described by Tost and Gut [32]. Pyrosequencing assays containing two PCR primers and one sequencing primer were designed to target CpG sites of interest using PyroMark Assay Design Software (Qiagen). One of the PCR primers had a universal tag which annealed to the universal biotinylated primer. Genomic DNA was sodium bisulfite converted the same way as for the Illumina microarray and amplified using Hot-Start Taq-polymerase (Qiagen). Amplicons were analyzed on a Q24 pyrosequencer (Qiagen) as specified by the manufacturer; % of methylation was quantified as the ratio of C to C + T using PyroMark Q24 Software (Qiagen). Pyrosequencing primers are shown in Additional file 1: Table S2.

### Quantitative real-time RT-PCR

To analyze tissue specific expression of *FBXL5*, *SCMH1* and *CACYBP* we performed real-time RT-PCR expression analysis. Total RNA was purchased from Clontech for 8 tissues and 4 brain regions. Lymphoblastoild cell line established from control sample C2 RNA was extracted using RNeasy kit (Qiagen). The cDNA for quantitative real-time PCR was synthesized using VersoTM cDNA kit (Thermo Fisher Scientific, Epsom, UK). Mx3005P QPCR System (Agilent, Santa Clara, CA) with SsoFast™ EvaGreen® Supermix (Bio-Rad, Hercules, CA). Expression of each gene was determined using the comparative Ct method and normalized to the expression of the *GAPDH* housekeeping gene. Primers sequences are shown in Additional file 1: Table S2.

## Results

### Methylation microarray profiling in patients with *KDM5C* mutations

To determine whether altered genome-wide DNA methylation patterns are associated with *KDM5C* mutations, we performed genome-wide DNA methylation analyses using the Illumina Infinium HumanMethylation27 BeadChip array containing 27,578 CpG sites and covering >14,000 genes in DNA from blood samples of XLID patients and controls. Our study group was comprised of 10 XLID patients from 5 families with 5 different *KDM5C* mutations. Two of these mutations were frameshift mutations, resulting in premature stop codons and 3 were missense mutations, predicted to be damaging by both Polyphen and Sift algorithms [4,5]. The number of affected individuals per family ranged from one to three. The control group was comprised of 16 age and ethnicity matched normal unrelated males and three unaffected male relatives (not carrying the mutation) from the family with the p.V504M mutation. We also tested two brothers with ID who carried a p.R1546Q sequence variant of unknown significance (VUS), predicted to be benign/tolerated by Polyphen and Sift respectively. During the course of this study, this *KDM5C* variant was reclassified as a benign variant, as it was found in a phenotypically normal maternal grandfather in another XLID family*.* Clinical and demographic information for the XLID patients and controls and locations of the *KDM5C* mutations are summarized in Additional file 1: Table S1 and Figure 1.

Methylation ratios (beta-values) for each of 27,578 CpG sites were calculated as ratios of methylated CpG to the sum of methylated and unmethylated CpG. The reliability of Illumina Infinium data was demonstrated by high correlation (R^2^ = 0.95-0.99) among samples as well as high correlation of microarray data with bisulfite pyrosequencing (R^2^ = 0.77-0.98) (Additional file 2: Figure S1).

Cross-reactive probes and probes overlapping SNPs in the queried CpGs [30] were excluded, leaving 23,837 sites for statistical analysis. As a first step in our exploratory analysis, we used principal component analysis (PCA) and unsupervised hierarchical clustering to create a high-level summary of the data for all 23,837 CpG sites. Examination of these results did not reveal a clear separation between the mutation cases, VUS cases, unaffected relatives, and controls (Additional file 2: Figures S2 - S3). This suggests that if there are DNA methylation differences associated with *KDM5C* mutations they occur at specific loci but not across all CpG sites analyzed.

To identify the differentially methylated CpG sites, we compared beta-values between 10 *KDM5C* mutation cases and 19 controls using the non-parametric Mann–Whitney *U* test. After generating an initial p-value for each of the 23,837 CpG sites, we observed that as many as 6,124 sites in a given sample were below the 0.05 significance level. To adjust for multiple testing we used a permutation-based method that estimated false discovery proportion (FDP) levels for pre-specified confidence levels [31]. We generated 1000 random permutations of the mutation labels among the data cases, while maintaining the sample sizes (10 vs. 19), and have identified significant CpG sites at four FDP levels for three confidence levels (Additional file 1: Table S3). The top candidates (53 CpG sites from 51 genes) with the lowest FDP = 0 and the highest confidence level of 99.5% (unadjusted p-values < 2.00e-07) are shown in Additional file 1: Table S4.

To address the potential effect of relatedness on differences in DNA methylation and controls we performed additional analysis including only unrelated individuals by randomly selecting 5 mutation cases from each of the five available families and 17 unrelated controls (one randomly chosen unaffected relative and 16 unrelated controls). To identify differentially methylated CpG sites, we performed the Mann–Whitney *U* test on all 72 possible combinations of 5 cases vs. 17 controls. We observed a large degree of consistency between the CpG sites identified in this analysis (5 unrelated cases vs. 17 unrelated controls) with the CpG sites identified in the general setting (10 cases vs. 19 controls). For example, the CpG sites found to be significantly different between cases and controls across all 72 combinations at 95% confidence level exactly coincided with the 53 CpG sites shown in Additional file 1: Table S4 (FDP =0 at 99.5% confidence in the complete dataset of 10 cases vs. 19 controls).

**Figure 2:**
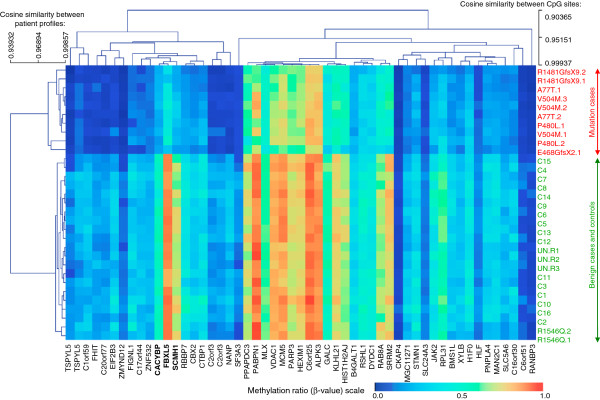
**Hierarchical unsupervised clustering of 31 study samples.** DNA methylation levels at the 53 most significant CpG sites were used for hierarchical unsupervised clustering. The results show a clear distinction between the 10 *KDM5C* mutation cases (top part), and the remaining 21 samples (bottom part), which is also indicated by the large distance between the two branches of the dendrogram on the left. DNA methylation levels corresponding to mutations are generally lower than for the controls. Interestingly, the two cases of variant of unknown clinical significance (p.R1546Q) have methylation profiles very similar to the 19 controls. Also, mutation cases from the same family do not always cluster together. The clustering was based on complete linkage and cosine distance metric.

We further tested if DNA methylation differences at 53 CpG sites with p-values < 2.00e-07 could differentiate cases either from controls or from the variant of unknown significance (p.R1546Q). A variety of unsupervised methods including hierarchical clustering (Figure 2), principal component analysis (Additional file 2: Figure S4), and K-means and K-median clustering (not shown) were capable of unambiguously differentiating *KDM5C* mutation cases from controls. Two samples, carrying the p.R1546Q variant at the C-terminal end of the KDM5C protein, consistently showed the same DNA methylation levels as control samples confirming the benign nature of this variant.

Multiple studies have shown that at some genomic loci DNA methylation levels could be affected by sequence polymorphisms in *cis* and subsequently be heritable [29,33-36]. We determined whether DNA methylation of the top 53 CpG sites were dependent not only on *KDM5C* mutation but also on single nucleotide polymorphisms (SNPs). For these analyses, we took advantage of two published datasets that used the same Illumina HumanMethylation27 microarray platform and reported a number of methylation quantitative trait loci (mQTL) in lymphoblastoid cell lines (LCLs) [33,34]. At these mQTLs DNA methylation depends on genotypes of single nucleotide polymorphisms (SNPs) located in *cis* or *trans* in relation to CpG. We did not find any mQTLs at the 53 CpG sites identified in our study, suggesting that the DNA methylation alterations we observed are more likely to be associated with *KDM5C* mutations rather than other genetic variation that exists between cases and controls.

**Figure 3:**
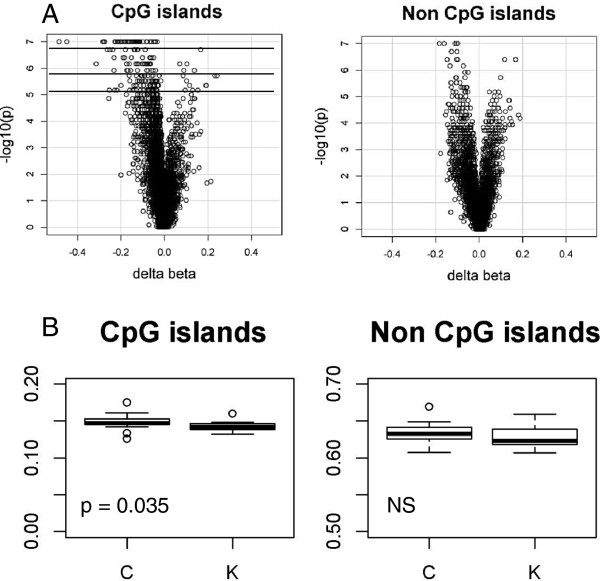
**Loss of DNA methylation at CpG islands associated with *KDM5C *mutations. A**) Volcano plot of DNA methylation differences between controls and *KDM5C* mutation cases (delta beta, X-axis) versus statistical significance –log10(p) in CpG islands (left) and non-CpG islands (right). Negative delta beta represents loss and positive delta beta gain of DNA methylation in *KDM5C* mutation cases. Black lines represent p-values at three levels of confidence 95% (6.69E-06), 99% (1.07E-06) and 99.5% (2.00e-07). **B**) Boxplot of the mean methylation of all microarray probes within CpG islands (left) and non-CpG islands (right) in controls (C) and in* KDM5C* mutations cases (K).

In agreement with the prediction of cross-talk between H3K4 methylation, and DNA methylation, we observed an over-representation of CpG sites with loss of DNA methylation versus gain at all levels of FDP and at all confidence intervals tested (Additional file 1: Table S3, p < 2.2e^-16^, Fisher Exact test). All 53 top CpG candidates with the lowest FDP = 0 and the highest confidence level of 99.5% (unadjusted p-values < 2.00e-07) exhibited loss of DNA methylation. The 13 CpGs with the greatest loss of DNA methylation (delta beta ≤-0.2) are shown in Table 1. In addition, in the volcano plot where delta beta is plotted against p-values, we observed an enrichment of CpG sites with negative delta beta, reflecting the loss of DNA methylation. Interestingly, this asymmetry was limited to CpG islands (Figure 3A). Also, averaging all microarray probes and comparing cases and controls we observed a small (<1%) loss of DNA methylation in the *KDM5C* mutation group (p = 0.035) also for CpG islands, but not for non-CpG island probes (Figure 3B). This genome-wide loss of DNA methylation was observed only in unique sequences, but not at LINE-1, the most common non-LTR retrotransposon, comprising about 17% of the human genome [37] as determined by pyrosequencing (Additional file 2: Figure S5).

**Table 1:** **Top 13 CpG sites with loss of DNA methylation in *KDM5C *mutation cases with the lowest false discovery proportion level =0, the highest confidence level of 99.5**% **and delta beta ≤ −0.2**

**Target ID**	**KDM5C AVG Beta**	**Control AVG Beta**	**VUS AVG Beta**	**p-value**	**delta beta**	**delta Z**	**SYMBOL**	**DISTANCE TO TSS**
**cg02630888**	0.41	0.89	0.91	9.98E-08	−0.48	2.03	FBXL5	−807
**cg03387723**	0.26	0.71	0.73	9.98E-08	−0.45	2.01	SCMH1	−676
**cg16743289**	0.24	0.52	0.53	9.98E-08	−0.28	2.02	CACYBP	−427
**cg06736444**	0.50	0.78	0.77	9.98E-08	−0.28	1.90	SRRM2	−861
**cg22809047**	0.31	0.59	0.54	9.98E-08	−0.27	1.91	RPL31	−490
**cg19884658**	0.55	0.77	0.74	9.98E-08	−0.23	1.98	KLHL21	−1,339
**cg14719055**	0.20	0.42	0.37	9.98E-08	−0.23	1.92	RBBP7	−1,218
**cg16604218**	0.09	0.31	0.35	9.98E-08	−0.22	1.84	EIF2B3	−349
**cg20318748**	0.07	0.29	0.18	9.98E-08	−0.22	1.95	NANP	−564
**cg03621001**	0.46	0.67	0.77	9.98E-08	−0.21	1.91	RAB8A	−771
**cg00328227**	0.08	0.28	0.30	9.98E-08	−0.2	1.95	C1orf59	−177
**cg05072008**	0.13	0.33	0.37	9.98E-08	−0.2	1.86	FIGNL1	−599
**cg03221914**	0.50	0.69	0.70	9.98E-08	−0.2	1.82	HIST1H2AJ	−813

VUS is p.R1546Q variant of unknown significance, AVG beta is mean beta value for each of three groups, delta beta is differences between mean of *KDM5C* mutations cases (N = 10) and controls (N = 19). Delta Z is delta beta divided by standard deviation of combined cases and controls data. All 13 CpG sites were located within CpG islands.

**Figure 4:**
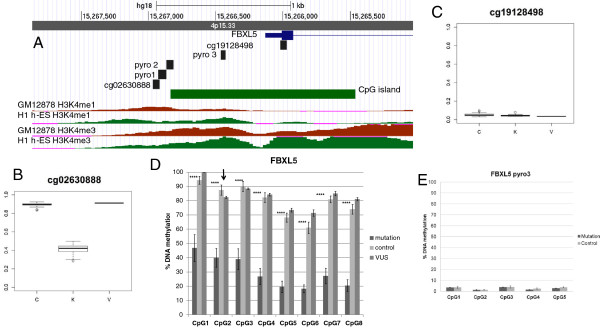
**Regional DNA methylation in *FBXL5 *promoter. A**) Screenshot from the UCSC genome browser showing location of *FBXL5* promoter exon1, intron1, CpG island, Illumina microarray probes and pyrosequencing assays and tracks for H3K4me1 and H3K4me3 in lymphoblastoid cell line (GM12878) and ES cell line (H1 h-ES) (Broad Institute Histone data). **B**&**C**) Boxplots of Illumina DNA methylation data for two microrray probes within *FBXL5* gene. The Y axis shows DNA methylation levels presented as C/C + T and ranging from 0 to 1. The bottom and the top of the box are 25th and 75th percentiles respectively; the whiskers are within the 1.5 interquartile range (IQR) of the data, and the circles, are outlier data points above or below 1.5 IQR. C are controls (N = 19), K are cases with *KDM5C* mutations (N = 10), V are cases with p.R1546Q sequence variant. **D**) DNA methylation upstream of *FBXL5* transcription start site as determined by pyrosequencing assays 1&2 (pyro1&2). The order of CpG sites are shown from the furthest upstream to the transcription start site. Each column is an average for each of three groups of 1)10 cases with *KDM5C* mutations (mutation), 2)19 controls (control), and 3) two individuals with the p.R1546Q variant of unknown significance (VUS). The arrow shows the CpG sites overlapping the microarray probe. P-values were determined by Kruskal-Wallis test between mutation cases and controls. **** is p <0.0001. **E**) DNA methylation upstream of *FBXL5* transcription start site and downstream of pyrosequencing assays 1&2 as determined by pyrosequencing assay 3 (pyro 3). The order of CpG sites are shown from the furthest upstream to the transcription start site. Each column is an average for each of two groups of 1) 5 cases with *KDM5C* mutations (mutation), 2) 5 controls (control). Error bars are standard deviations.

Illumina HumanMethylation27 coverage on average extends to two CpG sites per gene. Of the 53 most significant CpG sites based on permutation analysis, only two genes *C2orf3* (*GCFC2*) and *TSPYL5* had two CpG sites meeting the permutation p-value cut off. In order to assess the genomic extent of the DNA methylation changes for each significant gene we have evaluated DNA methylation levels, delta beta differences, and p-values in cases and controls for additional array probes for the 53 top significant genes. For a majority of the genes the second probe did not exhibit significant loss of DNA methylation. Only 8 genes had a second significant probe with p ≤ 0.05. The absolute delta beta differences for the second probes were relatively small ≤ 0.05, with the exception of the *STMN1* gene having a delta beta −0.16 and −0.14 in two CpG sites (Additional file 1: Table S5). The CpG sites with significant loss of DNA methylation tended to be located just on the edge of CpG island several hundred base pairs upstream of TSS. In contrast, CpG sites without DNA methylation differences were predominantly unmethylated in both cases and controls and located a few 100 bp downstream of TSS (Additional file 1: Table S5, Figure 4 and Additional file 2: Figures S6 - S9).

### Targeted validation by pyrosequencing

We have previously shown that validation of Illumina HumanMethylation27 CpG methylation by bisulfite pyrosequencing demonstrates high correlation between the two methods and methylation determined by Illumina microarray is a good predictor of regional CpG methylation in CpG islands [38]. To validate the DNA methylation differences found on the array we tested five loci: top three candidates *FBXL5* (delta beta = −0.48), *SCMH1* (delta beta = −0.45) and *CACYBP* (delta beta = −0.28*)* with the largest DNA methylation differences and two loci with smaller differences *DYDC1* (delta beta = −0.1) *ZMYND12* (delta beta = −0.08). Using pyrosequencing, we validated the direction of the differences for all 5 loci and observed high correlation DNA methylation levels between pyrosequencing and Illumina for overlapping CpG sites (R^2^ ranging from 0.77 to 0.98, Additional file 2: Figure S1). For *FBXL5*, *SCMH1, CACYBP* and *ZMYND2* the assays contained >1 CpG site, and pyrosequencing data showed that DNA methylation changes affected not only the index CpG site from the array but also multiple adjacent CpGs (Figures 4 and Additional file 2: Figures S6-S9). The samples carrying the p.R1546Q variant consistently exhibited the same DNA methylation patterns as controls. For *FBXL5* (8 CpGs) and *ZMYND2* (5 CpGs) all sites tested exhibited DNA methylation differences between cases and controls (Figures 4, Additional file 2: Figure S8). For *SCMH1* (5 CpGs), the CpG sites more distant from the TSS exhibited overall higher DNA methylation in controls and larger differences in DNA methylation between cases and controls (Additional file 2: Figure S6). For *CACYBP,* it was not possible to design a pyrosequencing assay overlapping the Illumina CpG site. Therefore, we designed an assay covering 2 CpGs ~100 bp upstream of the Illumina site. In this assay one CpG site exhibited significant DNA methylation differences between cases and controls consistent with the difference found on the array (Additional file 2: Figure S7).

For *FBXL5* we designed an additional assay to test DNA methylation within a CpG island in closer proximity to the TSS. We found very low overall DNA methylation levels in both cases and controls (<10%) with no significant differences. Interestingly, based on ChIP sequencing data for histone marks from the Broad Institute [39], sites that were hypermethylated in controls and exhibited significant loss of DNA methylation in *KDM5C* mutations cases frequently coincided with enhancer mark H3K4me1 in embryonic stem cell (ES) and lymphoblastoid cell lines. In contrast, the mark of active promoters H3K4me3 was shifted more towards the TSS, where DNA was hypomethylated (Figure 4, Additional file 2: Figures S6-S9). Thus, it is possible that sequences exhibiting loss of DNA methylation in patients with *KDM5C* mutations are involved in the regulation of downstream genes through enhancer activity.

### Ubiquitous expression of *FBXL5*, *CACYBP* and *SCMH1* in human tissues

We focused the next phases of our downstream analysis on the three top candidate genes *FBXL5, SCMH1* and *CACYBP* with the highest delta Z scores (Table 1). Interestingly, the three top CpG sites identified by methylation array to be significantly hypomethylated in *KDM5C* mutations cases are within promoters of genes involved in ubiquitin-mediated protein degradation [40-43]. FBXL5 is an iron sensing E3 ubiquitin ligase that regulates iron homeostasis [44]. CACYBP is part of a ubiquitin ligase complex regulating beta-catenin, which is important for cell-cell adhesion and transcription regulation through Wnt-signaling [45]. SCMH1 is part of a polycomb group complex 1 (PcG1) involved in transcriptional silencing [46] and proteosomal degradation for the Geminin protein, important for regulation of replication and maintenance of undifferentiated states [42]. Little is known however about tissue specific expression of these genes in human. We tested the expression of *FBXL5*, *SCMH1* and *CACYBP* in several somatic tissues including brain, kidney, heart, muscle and lymphoblastoid cells, as well as several brain regions. We observed ubiquitous expression across tissues and brain regions (0.6-12% of *GAPDH* expression level) (Additional file 2: Figure S10), consistent with the function of these genes in pathways important for multi-systemic physiological processes.

### Loss of DNA methylation associated with *KDM5C* mutations is not due to altered blood cell counts

There are no reported blood cell-related phenotypes associated with *KDM5C* mutations [7]. However, white blood cells consist of functionally distinct cell populations in varying proportions and it has been shown that at some loci in different white blood cells DNA methylation patterns can vary substantially [47]. As we did not have blood cell counts for either cases or controls in our study, the possibility that observed DNA methylation differences could be due to differences in the proportion in different cell types cannot be completely ruled out. To test this, we checked DNA methylation levels for the three top candidates *FBXL5*, *SCMH1* and *CACYBP* using GEO dataset (GSE35069). This dataset analyzed genome-wide DNA methylation using Illumina Methylation450 array in DNA from whole blood, peripheral blood mononuclear cells, and granulocytes, as well as in 7 isolated cell populations (CD4+ T cells, CD8+ T cells, CD56+ NK cells, CD19+ B cells, CD14+ monocytes, neutrophils, and eosinophils) in six healthy males [47]. The beta values for probes overlapping Illumina27 with significant loss of DNA methylation in *KDM5C* mutations cases were extracted for analysis. We did not observe differences between cell types at three CpG sites analyzed at a magnitude that could explain loss of DNA methylation in *KDM5C* mutations cases (Additional file 2: Figure S11). For *FBXL5*, DNA methylation was very uniform across all cell types, with a maximum difference of 2%, for *SCMH1* and *CACYBP* the biggest differences were 22% between CD19+ B cells and CD4+ T cells and 13% for CD19+ B cells and neutrophils, respectively. This comparison strongly suggests that the observed loss of DNA methylation found in individuals with *KDM5C* mutations are not due to differences in the proportion of blood cell types.

### DNA methylation patterns at *FBXL5, SCMH1* and *CACYBP* promoters in general population

**Figure 5:**
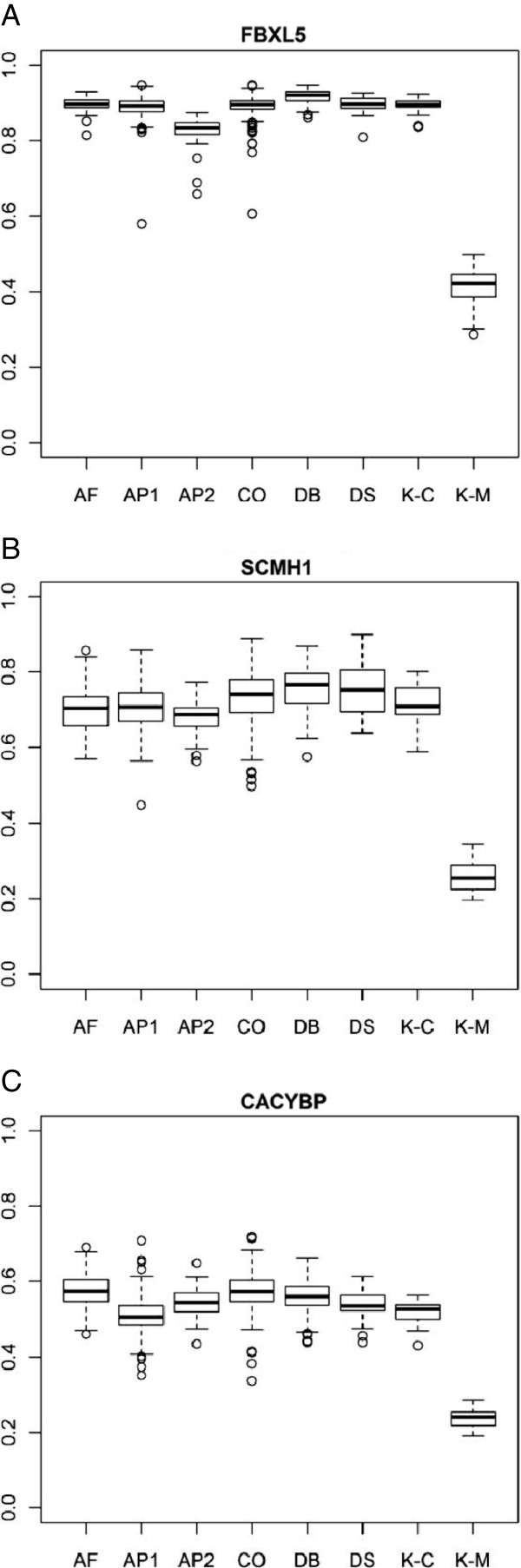
**DNA methylation at the three top significant CpGs in *KDM5C *mutations cases and population controls.** DNA methylation microarray data at three CpG sites within CpG-rich promoters of three genes *FBXL5* (**A**), *SCMH1* (**B**) and *CACYBP* (**C**) as determined in 6 published studies using Illumina methylation27 array. AF (Aging in females, n = 93), AP1 (aging pediatric 1, n = 398), AP2 (aging pediatric 2, n = 79), CO (cancer ovarian, n = 257), DB (diabetes, n = 99), DS (Down syndrome, n = 21), K-C are controls from our study (N = 16), K-M are *KDM5C* mutations cases. For CO and DS only control samples were included.

In our discovery dataset of 10 *KDM5C* mutations cases and 19 controls, we observed no overlap in DNA methylation levels between cases and controls for three genes *FBXL5*, *SCMH1* and *CACYBP*. We wanted to test the frequency of loss of DNA methylation in these three genes in the general population. For these analyses we used 6 Illumina Infinium HumanMethylation datasets of white blood cells from GEO NCBI database, comprising a total 946 control samples passing QC (Additional file 1: Table S6). Three datasets (GSE36064, GSE27097, GSE20236) [48,49] investigated DNA methylation association with age and included only control samples, while the other studies investigated DNA methylation association with disease including diabetes/nephropathy (GSE20067) [27], ovarian cancer (GSE19711) [27] and trisomy 21 (GSE25395) [50]. Due to common nature of diabetes we have included samples with this disease into our control dataset, but have excluded ovarian cancer samples because cancer as well as cancer therapies are known to alter epigenetic marks [51] and somatic *KDM5C* mutation was previously identified in cancer [52,53]; trisomy 21 cases were also excluded due to overlapping ID phenotype with *KDM5C* mutations cases [50]. None of the 946 control samples exhibited loss of DNA methylation comparable with *KDM5C* mutations cases (Figure 5).

**Figure 6:**
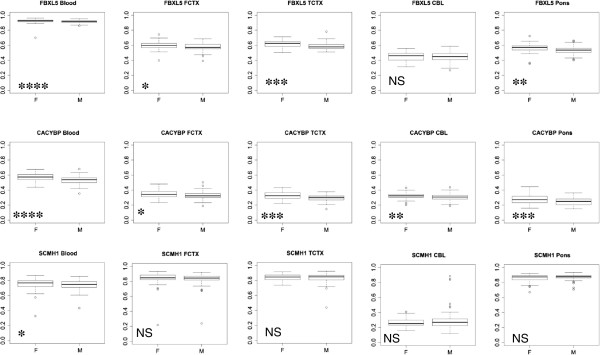
**Sex-specific DNA methylation differences in *FBXL5, CACYBP *and *SCMH1 *promoters in blood and four brain regions.** Boxplots show DNA methylation levels in three CpGs located in the promoters of *FBXL5*, *CACYBP* and *SCMH1* in 99 (48 males/51 females) blood samples (GSE20067), 133 (90 males/43 females) frontal cortex samples (FCTX), 127 (85 males/42 females) temporal cortex samples (TCTX), 121 (86 males/35 females) cerebellum samples (CBL) and 125 (87 males/38 females) pons samples from neurologically normal individuals (GSE15745). The Y axis shows DNA methylation levels presented as C/C + T and ranging from 0 to 1. The bottom and the top of the box are 25th and 75th percentiles respectively, the whiskers are within the 1.5 interquartile range (IQR) of the data, and the circles, are outlier data points above or below 1.5 IQR. P-values were calculated using Kruskal-Wallis test. **** is p <0.0001, *** is p < 0.001, ** is p < 0.01, * is p < 0.05, NS is p > 0.1.

We have also analyzed the association of DNA methylation levels at these sites with age, ethnicity, and sex for the 6 datasets. These data were analyzed separately within each study to avoid possible batch effects. We did not observe any significant association of DNA methylation at these three loci with ethnicity or age. There was a small (median differences = 0.01-0.04) but highly significant increase of DNA methylation in females compared to males in *FBXL5* and *CACYBP* (p < 1.00E-04) and a trend towards significance for *SCMH1* (p = 0.07), in the diabetes study where samples of both sexes were included [27] (Figure 6). We also assessed the consistency of the observed sex-specific differences in 9 additional autosomal CpG sites (top candidates with the largest DNA methylation loss, delta beta ≤ −0.2 from Table 1). Similar to differences described for *FBXL5, CACYBP* and *SCMH1,* we observed a significant (q-value ≤ 0.05) increase of DNA methylation in females compared to males in 5 loci and a trend towards significance (q-value ≤ 0.1) in three out of 9 tested loci with the delta beta differences ranging from 0.01 to 0.06 (Additional file 1: Table S7).

### DNA methylation comparison at *FBXL5, SCMH1* and *CACYBP* between brain and blood

Since brain tissue from individuals with *KDM5C* mutations is not available for study, we took an alternative approach to assess whether the genomic targets we identified might be demonstrated to be functionally important in brain. In this regard, we investigated whether DNA methylation levels in brain are similar to those in blood and whether sex-specific DNA methylation differences we found in blood are also observed in the brain. For this analysis we used a published Illumina HumanMethylation27 dataset for four brain regions (temporal cortex, frontal cortex, cerebellum and pons) of neurologically normal individuals (GEO Accession No: GSE15745) [29]. DNA methylation at three tested CpG sites exhibited overall hypermethylation (methylation level > 50%) in brain and blood for *FBXL5* and *SCMH1* with the exception of cerebellum which had intermediate methylation levels in brain (30-40%) and high methylation levels in blood (70%). *CACYBP* had intermediate levels of DNA methylation in both brain and blood (30-50%) (Figure 6). Furthermore, we observed a small but statistically significant increase of DNA methylation in females compared to males in three brain regions at *FBXL5* and in four brain regions at *CACYBP*, which was similar to sex-specific difference found in blood (Figure 6, Additional file 1: Table S8). In conclusion, these data suggest that DNA methylation at *FBXL5* and *CACYBP* can be regulated by similar mechanisms in both blood and brain and the observed sex- specific differences could be the result of differences in *KDM5C/KDM5D* dosage between males and females.

### DNA methylation levels at *FBXL5*, *SCMH1* and *CACYBP* and *KDM5C/KDM5D* dosage in blood

**Figure 7:**
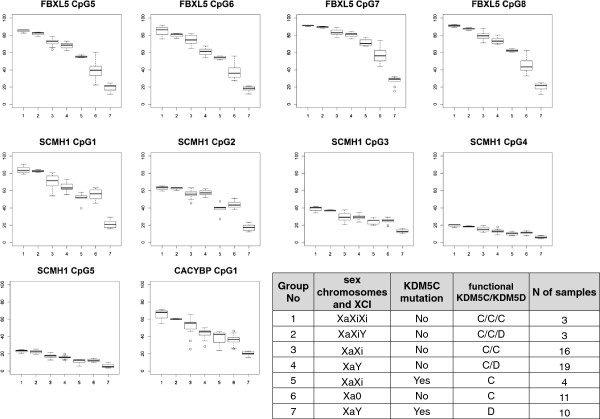
**DNA methylation at *FBXL5, SCMH1 *and *CACYBP *promoters correlates with *KDM5C/KDM5D *dosage.** Boxplots show DNA methylation levels for CpG sites within the *FBXL5, SCMH1* and *CACYBP* promoters in blood in 7 group of samples with different dosage of *KDM5C/KDM5D*. CpG numbering corresponds to Figures 4D, 6D and 7D. *CACYBP* CpG#2 boxplot is not shown, as no correlation with *KDM5C/KDM5D* dosage was found for this site. The Y axis is % of DNA methylation. The X axis shows groups of samples, numbered from 1 to 7. Information for each group regarding sex chromosome constitution, X-chromosome inactivation (XCI, Xa is active and Xi is inactive X-chromosome respectively), presence/absence of *KDM5C* mutation, functional *KDM5C/KDM5D* dosage, and number of samples is shown in the table beside the graph.

*KDM5C* is an X-linked gene that escapes X-inactivation in humans and mouse [18,19,54], and has a functional Y-linked homologue *KDM5D*[55]. Interestingly, in mouse the degree of *Kdm5c’s* escape from X –inactivation is highly variable across different tissues. The level of transcript from the inactive X allele is 20-70% of the active X allele [56]. It is not known if the same variability is present in humans. In mouse brain, *Kdm5c/Kdm5d* are expressed in a sex-specific fashion i.e., the expression of *Kdm5c* is significantly higher in female brains than in male brains, and the expression of *Kdm5d* in males is not sufficient to compensate for the female bias in *Kdm5c* expression [21]. Furthermore, in human tissues, *KDM5D* is reported to be expressed at lower levels than *KDM5C*. However, as commercially available RNA mixed from several individuals was used for this experiment the proportion of male cells present in these samples is not known [3]. Our observation of increased DNA methylation in females compared to males in the top three affected loci (Figure 6), led us to hypothesize that DNA methylation at these loci might depend on sex chromosome dosage and specifically on the dosage of the X and Y linked homologues, *KDM5C* and *KDM5D,* respectively. To further investigate this, we assessed *FBXL5, SCMH1* and *CACYBP* DNA methylation levels using targeted pyrosequencing assays in blood samples with different sex chromosome constitutions and reflecting variation in *KDM5C*/*KDM5D* dosage, including 47,XXX (*KDM5C/KDM5C/KDM5C*, N = 3), 47,XXY (*KDM5C/KDM5C/KDM5D*, N = 3), 46,XX (*KDM5C*/*KDM5C*, N = 16), 46,XY (*KDM5C*/*KDM5D*, N = 19) and 45,X (KDM5C/0, N = 11) in comparison to males with *KDM5C* mutations (*0/KDM5D*, N = 10) and female carriers of *KDM5C* mutation (*KDM5C/0*, N = 4). We found that the DNA methylation levels at these three genes generally correlated with *KDM5C/KDM5D* dosage for all three genes analyzed (Figure 7). 47,XXX females exhibited the highest DNA methylation closely followed by 47, XXY males, 46,XX females and 46,XY males for the majority of analyzed CpG sites. There were less differences for *SCMH1* between 46,XX females and 46,XY males suggesting that for this gene other factors might be involved in equalizing DNA methylation between the two sexes.

DNA methylation levels were similar for 45,X females and female carriers of *KDM5C* mutations but significantly lower than in the four groups described above. This suggests that a single functional copy of *KDM5C,* without additional activity from *KDM5D,* is not sufficient to achieve levels of DNA methylation present in females and males with normal karyotypes. Males with *KDM5C* mutations exhibited the lowest DNA methylation of the 7 analyzed groups. They had significantly lower DNA methylation than females with only one functional copy of *KDM5C* (45,X and *KDM5C* mutation female carriers) (Figure 7), suggesting that *KDM5D* alone is not sufficient to compensate for absence of functional the *KDM5C*.

The differences among groups with normal or extra copies of *KDM5C/KDM5D* (the 47,XXX, 47, XXY, 46, XX and 46, XY) were substantially smaller than the differences for cases missing one copy of functional *KDM5C (*45, X, females and males with *KDM5C* mutations). This could be due to the fact that two copies of *KDM5C* or one copy each of *KDM5C* and *KDM5D* is close to saturation of H3K4 demethylase activity at target promoters. Another possible explanation of small differences between 46, XX females and 46, XY males observed in both brain and blood, is that *KDM5D* is expressed only at slightly lower levels than *KDM5C* from inactive X-chromosome in human, making 46,XX and 46,XY relatively close in their levels of H3K4 demethylase activity, in contrast to larger differences between males with *KDM5C* mutation vs. 45,X females, where the difference is between *KDM5D* and *KDM5C* expressed from an active X. The observation that *KDM5C* mutation female carriers exhibit DNA methylation levels similar to individuals with 45,X karyotype and the fact these females have highly skewed X-chromosome inactivation [5], suggests that in carriers the wild type *KDM5C* is expressed from the preferentially active X-chromosome.

Based on these data we suggest that DNA methylation levels at *FBXL5, SCMH1* and *CACYBP* promoters correlate with H3K4 demethylase activity of the proteins KDM5C/KDM5D due to an inverse relationship between H3K4 methylation and DNA methylation [22].

## Discussion

Advances in molecular technologies have helped to identify genetic causes in many cases of syndromic and non-syndromic forms of intellectual disability (ID) [57-59]. However, the molecular pathogenesis of ID still remains incompletely understood. It has been suggested based on known genetic etiologies that perturbed neuronal homeostasis altering synaptic outputs could be a key component of the cognitive impairment phenotype [60]. This notion is strongly supported by the types of functions attributable to genes mutated in ID which include basic cellular functions, such as transcription, translation, RNA biogenesis, protein turnover, and cytoskeletal dynamics [60]. One important emerging mechanism in ID is epigenetic dysregulation that ultimately affects transcription of multiple genes [1,57]. KDM5C is one of more than 20 epigenetic regulators involved in ID [1]. The identification of specific downstream targets exhibiting aberrant epigenetic marks in response to mutation of an epigenetic regulator will have an important impact on our understanding of the molecular pathogenesis of ID.

Previously, profiling of mRNA in lymphoblastoid cell lines of 12 males with *KDM5C* mutations compared to 5 controls identified 11 upregulated genes. These transcriptional changes were not very consistent among *KDM5C* mutations samples, and a combination of at least 6 genes was required to distinguish cases from controls [15]. None of these 11 genes exhibited DNA methylation changes in our dataset. Thus, it is likely that these expression differences are more tissue and developmental stage specific, than the DNA methylation patterns, potentially reflective of disrupted binding of KDM5C specifically in lymphoblastoid cell lines. DNA methylation patterns can be maintained by DNMT1 through replication and multiple cell divisions [61], and thus if they occur early in development they could be represented in multiple lineages including peripheral blood, but not be reflective of gene expression patterns in all differentiated lineages. This has, in fact, been observed in neurodevelopmental syndromes such as Immunodeficiency–centromeric instability–facial anomalies (ICF), Fragile-X, Angelman and Prader-Willi syndromes [62]. Further, the regions where we found loss of DNA methylation associated with *KDM5C* mutations, coincided with an enhancer mark H3K4me1 in ES and lymphoblastoid cell lines. In contrast, DNA sequences more proximal to the TSS were hypomethylated in both controls and cases and coincided with the active promoter mark H3K4me3 (Figure 4, Additional file 2: Figures S6-S9) [39]. These data suggest that regions affected by loss of DNA methylation have enhancer-driven rather than basal promoter function. As enhancers are involved in the control of spatial and temporal gene expression [63], the relationship between loss of DNA methylation at identified sites and expression at downstream genes is likely to be more complex than a simple inverse correlation.

The mechanism of the observed loss of DNA methylation associated with loss of function mutations in *KDM5C* is not completely clear; however, it is unlikely to be the direct consequence of loss of KDM5C function, as KDM5C is not known to possess DNA methyltransferase activity. Based on the current literature, the most plausible mechanism is that a deficiency in H3K4 demethylase activity leads to increased H3K4 methylation, which protects DNA from *de novo* DNA methylation at KDM5C downstream target loci. In mouse ES cell, Dnmt3L recruits *de novo* methyltransferases to DNA associated with unmethylated forms of H3K4, and contact between Dnmt3L and the nucleosome is inhibited by all forms of H3K4 methylation [26]. Biochemical assays have shown that the human *de novo* methyltransferase DNMT3A interacts with histone H3 unmethylated at K4, whereas di- and tri-methylation inhibit this interaction [64]. There is also evidence from a yeast model system lacking endogenous DNA methyltransferases and ectopically expressing mouse Dnmt3a and Dnmt3L that depletion of H3K4 methylation results in increased DNA methylation [65]. In humans, a correlation of increased tri-methylation in H3K4 with reduced DNA methylation at promoters has been shown in fibroblast cells from normal individuals [66].

Our data demonstrating DNA methylation alterations in individuals with mutations in the *KDM5C* gene further support the link between H3K4 methylation and DNA methylation. These data show, for the first time, a functional consequence of loss of function of H3K4 demethylase resulting in significant alterations of DNA methylation at specific gene targets at a genome-wide level. Furthermore, in agreement with cross-talk of H3K4 methylation and DNA methylation, there was significantly more loss than gain of DNA methylation resulting from mutations in *KDM5C*. In mammals several H3K4 demethylases have been described. Apart from four enzymes of the KDM5 family, KDM1A and B specifically act to demethylate di- and mono-methylated forms of H3K4, and KDM2B similarly to the KDM5 family specifically demethylates tri- and di-methylated forms of H3K4 [67,68]. At this point it is not clear if the loss of DNA methylation associated with *KDM5C* mutations is specific to KDM5C loss of function or would also be observed in the context of loss of function of other H3K4 demethylases. In support of the concept that loss of DNA methylation could be a common association of loss of H3K4 demethylase activity, mouse oocytes deficient in the H3K4 demethylase Kdm1b demonstrated a global increase in H3K4 di-methylation and failed to generate normal DNA methylation marks at several imprinted loci [68]. However, as we did not observe loss of DNA methylation at imprinted genes in patients with *KDM5C* mutations, the genomic sites demonstrating loss of DNA methylation for each H3K4 demethylase are likely to be specific, possibly reflecting the binding sites of these proteins.

Further support for the inter-dependence of histone methylation and DNA methylation in humans comes from cancer research. Mutations in *IDH1* and *IDH2*, frequently found in gliomas and acute myeloid leukemias (AML), are characterized by enzymatic gain of function and subsequent production of hydroxyglutarate, which inhibits several histone demethylases, including H3K9, H3K27, H3K36 and H3K4 [69]. The somatic mutations in *IDH1/IDH2* are associated with genome-wide hypermethylation in AML compared either to normal bone marrow or to AML caused by mutations in other genes. However at this point it is not clear which specific histone marks contribute directly to this DNA hypermethylation phenotype [70]. Interestingly, two genes, *PABPN1* and *ZNF532*, demonstrating loss of DNA methylation in our study (Additional file 1: Table S4) were found to be hypermethylated in AML with *IDH1* mutation [70]. These data suggest that there could be some common mechanism regulating DNA methylation of these two genes in opposite directions in the context of loss of function of *KDM5C* and gain of function of *IDH1*.

The genes *FBXL5*, *SCMH1* and *CACYBP* on which we focused in our downstream analysis have exhibited a surprisingly large degree of DNA methylation differences between cases and controls reminiscent of the DNA methylation alterations at imprinted loci in disorders affecting neurodevelopment, such as Prader-Willi and Angelman syndromes [71,72]. Further, loss of DNA methylation at these sites was not observed in 946 population control blood samples from publically available datasets, suggesting that altered DNA methylation at these three genes could be used for establishing pathological authenticity of new missense mutations, as *in silico* predictions of effects on protein function are often inconclusive, and functional experiments are expensive and labor intensive. Thus, as *KDM5C* mutation cases are frequently indistinguishable from other genetic causes of ID based on clinical phenotype alone [15], DNA methylation analysis could complement *KDM5C* sequencing to provide more accurate molecular diagnosis leading to improved patient management.

Interestingly, the three top candidate genes are part of ubiquitin-ligase protein degradation pathways. Synaptic network remodeling, a vital part of central nervous system function, depends on ubiquitin-mediated protein degradation at the postsynaptic membrane [73]. Genes involved in ubiquitination pathways have already been implicated in a number of other neurodevelopmental disorders, such as Angelman syndrome (loss of function of maternal copy of *UBE3A*) [71], and autism (copy number variants in *PARK2*, *RFWD2*, *FBX040*) [74]. Furthermore, 7% of XLID genes identified to date are components of the ubiquitin pathway [75].

We have shown that these three genes are ubiquitously expressed in human tissues at relatively low levels compared to the house-keeping gene *GAPDH*. Although it is currently not clear how loss of DNA methylation at these sites affect gene expression, we propose that abnormal expression of these genes at specific cell types/developmental stages causes disturbances in downstream pathways such as degradation of target proteins. FBXL5 has been recently discovered to be a component of an E3 ubiquitin ligase complex that targets IRP2, an iron regulatory protein 2 important in intracellular and plasma iron homeostasis [43,76]. IRP2 regulates RNA stability and translation by binding to iron responsive - RNA stem loop structures, in a number of genes involved in iron uptake, storage and utilization [44]. While FBXL5 loss of function leads to embryonic lethality in Fbxl5−/− mice, associated with aberrant iron accumulation and increased oxidative stress [77], Irp2 −/− knockout mice exhibit a neurological phenotype associated with locomotor abnormalities accompanied by iron accumulation in white and grey matter [78]. Thus, abnormal expression of *FBXL5* could result in abnormal iron accumulation, thereby contributing to seizures and/or ID phenotypes observed in males with *KDM5C* mutations. SCMH1 is a member of the Polycomb-group 1 complex, which not only is a transcriptional repressor, but also acts as an E3 ubiquitin ligase for the geminin protein involved in DNA replication and maintenance of undifferentiated cellular states. Specifically, SCMH1 has been shown to provide an interaction domain for geminin [42]. Recently a genome-wide association study implicated *SCMH1* in the regulation of human height [79], thus it is possible that loss of DNA methylation at *SCMH1* is important for short stature associated with *KDM5C* mutations. CACYBP (Sip) is part of the SCF-like complex, involved in ubiquitin-mediated degradation of the transcriptional activator β-catenin [40,41]. β-catenin is a signaling molecule playing an important role in neurodevelopmental processes such as neural crest development, development of cortical and hippocampal neuroepithelium, and dendrite spine morphogenesis [80-82]. In addition, it has been implicated in seizure susceptibility [83]. Further, CACYBP was shown to dephosphorylate ERK1/2 [84], extracellular signal-regulated kinases, important in many aspects of early brain development and implicated in 16p11.2 and 22q11 deletion syndromes phenotypes [85].

As the described DNA methylation changes in our study were identified in blood samples, an important question that cannot be directly addressed by our data is the issue of whether parallel changes occur in brain. As *de novo* DNA methylation is an important process in epigenetic reprogramming occurring at early stages of embryonic development [22,86], we suggest that loss of DNA methylation in the blood of patients with *KDM5C* mutations could at least in part result from abnormally high H3K4 di/trimethylation in the embryo, protecting DNA from *de novo* methylation. We expect that this state is maintained through differentiation into multiple lineages. In support of this, we found parallel sex-specific DNA methylation differences in both brain and blood at *FBXL5* and *CACYBP*, whereas *SCMH1* exhibited this difference only in blood, but not in brain. We suspect that these observed sex-specific differences are due to *KDM5C/KDM5D* dosage rather than the effects of sex hormones, as the DNA methylation at tested targets correlates better with sex chromosome constitution than with gonadal sex, e.g. the highest DNA methylation was observed in 47,XXX females, followed by 47,XXY males, 46,XX females, 46,XY males, 45,X females. The lowest DNA methylation is seen in males with *KDM5C* mutations (Figure 7). In addition we observed that female carriers of *KDM5C* mutations have DNA methylation levels similar to 45,X females, reflecting the fact that they have only one functional copy of *KDM5C*.

Based on DNA methylation comparison between brain and blood, we propose that the epigenetic status of *FBXL5* and *CACYBP* is regulated by KDM5C in both brain and blood, and that their deregulation in brain can contribute to the intellectual disability and seizure phenotypes in individuals with *KDM5C* mutation. We propose as well that KDM5C contributes to sex-specific differences in brain function. In contrast, *SCMH1* might be responsible for other aspects of the clinical phenotype associated with *KDM5C* mutation such as growth abnormalities. Furthermore, the *KDM5C-*mutation associated targets identified here could play a role in Turner syndrome. It has been previously suggested that X-linked genes escaping X-inactivation such as *KDM5C* are likely to be implicated in neurocognitive phenotypes of 45,X females with Turner syndrome, who in spite of normal cognitive abilities, frequently have problems in spatial reasoning and emotion recognition [87,88]. Our observation of loss of DNA methylation at the *FBXL5, SCMH1* and *CACYBP* promoters in 45, X females compared to XX females and XY males, but to a lesser degree than in males with *KDM5C* mutations, supports this hypothesis and suggests that deregulation of epigenetic targets of KDM5C could be relevant to the mild neurodevelopmental impairments found in females with Turner syndrome. Similarly, loss of DNA methylation at these three genes found in female carriers of *KDM5C* mutations could contribute to learning difficulties frequently observed in such individuals [5,7].

In summary these data provide new opportunities to address the molecular basis, both genetic and epigenetic, of ID. An important area for future investigation would be to establish both spatial (tissue-specific) and temporal (developmental stage- specific) maps of KDM5C targets, and to annotate how loss of *KDM5C* function impacts expression of these targets through embryonic development and in diverse tissues. Validation of the affected molecular pathways, described here such as abnormal iron homeostasis or β-catenin dysregulation could also, in an animal model of *KDM5C* mutations, provide a framework for potential therapeutic developments for patients with *KDM5C* mutations.

## Conclusions

We have, for the first time, identified significant multilocus loss of DNA methylation in individuals with loss of function mutations of a gene encoding histone modifying enzyme, specifically a histone H3K4 demethylase KDM5C. We have validated changes in three loci with the most prominent changes: *FBXL5, SCMH1* and *CACYBP*. We have also demonstrated that loss of DNA methylation at these three genes is specifically associated with *KDM5C* mutations and is not observed in >900 control blood samples. In addition we have shown that DNA methylation at these three genes correlates with dosage of *KDM5C* and its Y-linked homologue *KDM5D* in blood of individuals with different sex chromosome complements. Finally we observed parallel sex-specific differences in several brain regions for *FBXL5* and *CACYBP,* suggesting that these genes could play an important role in the ID phenotype of individuals with *KDM5C* mutations.

## Abbreviations

ID: Intellectual disability; XLID: X-linked intellectual disability; H3K4: Histone H3 Lysine 4; ES: embryonic stem; AML: acute myeloid leukemia.

## Competing interests

The authors declare that they have no competing interests.

## Authors’ contributions

Conceived and designed the experiments: DG, BHYC, DTB, RW. Performed the experiments: DG, BHYC, SJG, YL and CZ. Analyzed data BHYC, DG, AT, SJG, SC, RR, YAC. Provided materials/reagents/analysis tools/clinical data: FEA, CS, JS, CAB, JH, SW, SWS, CES. Wrote manuscript DG, BHYC, DTB, AT, SC, RW. All authors read and approved the final manuscript.

## Pre-publication history

The pre-publication history for this paper can be accessed here:

http://www.biomedcentral.com/1755-8794/6/1/prepub

## Supplementary Material

Additional file 1 **Table S1.** Clinical features and demographic information of the 10 male patients with X-linked intellectual disability due to KDM5C mutations, 2 male patients with a variant of unknown significance. **Table S2:** Primer Sequences. **Table S3:** Number of significant CpG sites with loss and gain of DNA methylation detected by multivariate permutation analysis for different levels of confidence (1-α) and false discovery proportion limit (γ). **Table S4:** The top 53 most significant CpG sites that are differentially methylated between the *KDM5C* mutations and normal controls with the lowest FDP = 0 and the highest confidence level of 99.5%. **Table S5:** The top 53 most significant CpG sites with additional CpG sites within the same genes. **Table S6:** GEO studies used to assess DNA methylation at at FBXL5, SCMH1 and CACYBP in blood samples of population controls. **Table S7:** Analysis of sex specific DNA methylation differences in blood samples from diabetes study (GSE20067). **Table S8:** Analysis of sex specific DNA methylation differences in brain in *FBXL5*, *SCMH1* and *CACYBP* promoters. Click here for file

Additional file 2 **Figure S1.** Scatter plots of DNA methylation determined by Illumina (X-axis) and pyrosequencing (Y-axis) at overlapping CpG sites. Grey diamonds are cases with *KDM5C* mutations, black squares are controls and grey triangles are individuals with p.R1546Q variant. **Figure S2:** Visual mapping of all 31 samples to the coordinate space defined by the first two principal components reveals that there is no clear separation between samples with and without *KDM5C* mutations. The principal component analysis (PCA) was performed using all 23,837 CpG sites. The red dots represent the 10 mutation cases, the 19 light green dots represent controls and the two dark green dots represent the benign mutation variants (p.R1546Q). **Figure S3:** Unsupervised hierarchical clustering of methylation data at 23, 837 CpG sites reveals that there is no clear separation between samples with and without KDM5C mutations. C1-16, are unrelated controls, UN-R1-2 are unaffected relative. R1546Q 1–2 are cases with benign variant, and the rest of the samples are KDM5C mutation cases. **Figure S4:** Visual mapping of all 31 samples to the coordinate space defined by the first two principal components. The principal component analysis (PCA) was performed using only the methylation levels at the 53 most significant CpG sites (the same CpG sites as shown in Figure 1). The red dots represent the 10 KDM5C mutation cases, the 19 light green dots represent controls, and the two dark green dots represent the benign mutation variants (p.R1546Q). Although the PCA procedure did not use any information on the mutation status, the data distribution shows a clear separation between aberrant mutations and benign mutations and controls. **Figure S5:** DNA methylation levels at 3 CpG sites in promoter of Long Interspersed Element-1 (LINE-1) as determined by pyrosequencing. The Y-axis is DNA methylation%. Two groups of samples, controls (C; N = 19) and *KDM5C* mutation cases (K;N = 10) are shown on the X-axis. **Figure S6:** Regional DNA methylation in *SCMH1* promoter. A) Screenshot from the UCSC genome browser showing location of *SCMH1* promoter exon1, intron1, CpG island, Illumina 27 K microarray probes and pyrosequencing assays and tracks for H3K4me1 and H3K4me3 in lymphoblastoid cell line (GM12878) and ES cell line (H1 h-ES) (Broad Institute Histone data). B&C) Boxplots of Illumina DNA methylation data for two microrray probes within *SCMH1* gene. The Y axis shows DNA methylation levels presented as C/C + T and ranging from 0 to 1. The bottom and the top of the box are 25th and 75th percentiles respectively, the whiskers are within the 1.5 interquartile range (IQR) of the data, and the circles, are outlier data points above or below 1.5 IQR. C are controls (N = 19), K are cases with KDM5C mutations (N = 10), V are cases with p.R1546Q sequence variant. D) DNA methylation upstream of SCMH1 transcription start site as determined by pyrosequencing assay (pyro). The order of CpG sites are shown from the further upstream towards the transcription start site. Each column is an average for each of three groups of 1)10 cases with *KDM5C* mutations (mutation), 2)19 controls (control), and 3) two individuals with the p.R1546Q variant of unknown clinical significance (VUS). The arrows show the CpG sites from the microarray. P-values were determined by Kruskal-Wallis test between mutation cases and controls. **Figure S7:** Regional DNA methylation in *CACYBP* promoter. A) Screenshot from the UCSC genome browser showing location of CACYBP promoter, exons1&2, introns 1&2, CpG island, Illumina 27 K microarray probes and pyrosequencing assays and tracks for H3K4me1 and H3K4me3 in lymphoblastoid cell line (GM12878) and ES cell line (H1 h-ES) (Broad Institute Histone data). B&C) Boxplots of Illumina DNA methylation data for two microrray probes within *CACYBP* gene. The Y axis shows DNA methylation levels presented as C/C + T and ranging from 0 to 1. The bottom and the top of the box are 25th and 75th percentiles respectively, the whiskers are within the 1.5 interquartile range (IQR) of the data, and the circles, are outlier data points above or below 1.5 IQR. C are controls (N = 19), K are cases with KDM5C mutations (N = 10), V are cases with p.R1546Q sequence variant. D) DNA methylation upstream of *CACYBP* transcription start site as determined by pyrosequencing assay (pyro). The order of CpG sites are shown from the further upstream towards the transcription start site. Each column is an average for each of three groups of 1)10 cases with *KDM5C* mutations (mutation), 2)19 controls (control), and 3) two individuals with the p.R1546Q variant of unknown significance (VUS). P-values were determined by Kruskal-Wallis test between mutation cases and controls. **** is p <0.0001, * is p <0.05. **Figure S8:** Regional DNA methylation in *ZMYMD12/PPCS* promoter. A) Screenshot from the UCSC genome browser showing location of *ZMYND12* and *PPCS* promoter, CpG island, Illumina 27 K microarray probes and pyrosequencing assays and tracks for H3K4me1 and H3K4me3 in lymphoblastoid cell line (GM12878) and ES cell line (H1 h-ES) (Broad Institute Histone data). B&C) Boxplots of Illumina DNA methylation data for two microrray probes within *ZMYND12* gene. The Y axis shows DNA methylation levels presented as C/C + T and ranging from 0 to 1. The bottom and the top of the box are 25th and 75th percentiles respectively, the whiskers are within the 1.5 interquartile range (IQR) of the data, and the circles, are outlier data points above or below 1.5 IQR. C are controls (N = 19), K are cases with KDM5C mutations (N = 10), V are cases with p.R1546Q sequence variant. D) DNA methylation upstream of *PPCS* transcription start site and within exon1/intrion1 of *ZMYND12* as determined by pyrosequencing assay (pyro). The order of CpG sites are shown from the further upstream towards the *PPCS* transcription start site. Each column is an average for each of three groups of 1)10 cases with *KDM5C* mutations (mutation), 2)19 controls (control), and 3) two individuals with the p.R1546Q variant of unknown significance (VUS). P-values were determined by Kruskal-Wallis test between mutation cases and controls. **** is p <0.0001. **Figure S9:** Regional DNA methylation in *DYDC1/DYDC2* promoter. A) Screenshot from the UCSC genome browser showing location of DYDC1/DYDC2 promoter, CpG island, Illumina 27 K microarray probes and pyrosequencing assays and tracks for H3K4me1 and H3K4me3 in lymphoblastoid cell line (GM12878) and ES cell line (H1 h-ES) (Broad Institute Histone data). B&C) Boxplots of Illumina DNA methylation data for two microrray probes within DYDC1 gene. The Y axis shows DNA methylation levels presented as C/C + T and ranging from 0 to 1. The bottom and the top of the box are 25th and 75th percentiles respectively, the whiskers are within the 1.5 interquartile range (IQR) of the data, and the circles, are outlier data points above or below 1.5 IQR. C are controls (N = 19), K are cases with KDM5C mutations (N = 10), V are cases with p.R1546Q sequence variant. D) DNA methylation upstream of *DYDC1* transcription start site as determined by pyrosequencing assay (pyro) overlapping Illumina CpG site cg17703212. Column is an average for each of three groups of 1)10 cases with KDM5C mutations (mutation), 2)19 controls (control), and 3) two individuals with the p.R1546Q variant of uknown significance (VUS). P-values were determined by Kruskal-Wallis test between mutation cases and controls. **** is p <0.0001. **Figure S10:** Expression of *FBXL5*, *SCMH1* and *CACYBP* in a panel of human tissues: BT -brain total, FB- fetal brain, CC-cerebral cortex, CB-cerebellum, TL- temporal lobe, FL –frontal lobe, SC –spinal cord, K-kidney, H-heart, L-liver, SM-smooth muscle, SK-skeletal muscle, LCL-lymphoblastoid cell line. Error bars are standard deviation of duplicated q-PCR experiments. **Figure S11:** DNA methylation levels of three CpG sites cg02630888 (*FBXL5*), cg03387723 (*SCMH1*), cg16743289 (*CACYBP*) in different blood cell types (GSE35069). The cell types are whole blood, peripheral blood mononuclear cells (PBMC), granulocytes and seven isolated cell populations (CD4+ T cells, CD8+ T cells, CD56+ NK cells, CD19+ B cells, CD14+ monocytes, neutrophils (Neu), and eosinophils (Eos)) which are each shown by different colour. Y-axis is beta methylation value determined by Illumina methylation450 array (GSE35069). Each column represents mean methylation from 6 samples from healthy males, error bars are standard deviation from the mean. Click here for file
