# Supplementary material for: Multilocus loss of DNA methylation in individuals with mutations in the histone H3 Lysine 4 Demethylase KDM5C
Source: BMC Med Genomics. 2013 Jan 28;6:1. doi: 10.1186/1755-8794-6-1 (PMC3573947; doi:10.1186/1755-8794-6-1)
Supplement: Additional file 2 — Figure S1. Scatter plots of DNA methylation determined by Illumina (X-axis) and pyrosequencing (Y-axis) at overlapping CpG sites. Grey diamonds are cases with KDM5C mutations, black squares are controls and grey triangles are individuals with p.R1546Q variant. Figure S2: Visual mapping of all 31 samples to the coordinate space defined by the first two principal components reveals that there is no clear separation between samples with and without KDM5C mutations. The principal component analysis (PCA) was performed using all 23,837 CpG sites. The red dots represent the 10 mutation cases, the 19 light green dots represent controls and the two dark green dots represent the benign mutation variants (p.R1546Q). Figure S3: Unsupervised hierarchical clustering of methylation data at 23, 837 CpG sites reveals that there is no clear separation between samples with and without KDM5C mutations. C1-16, are unrelated controls, UN-R1-2 are unaffected relative. R1546Q 1–2 are cases with benign variant, and the rest of the samples are KDM5C mutation cases. Figure S4: Visual mapping of all 31 samples to the coordinate space defined by the first two principal components. The principal component analysis (PCA) was performed using only the methylation levels at the 53 most significant CpG sites (the same CpG sites as shown in Figure 1). The red dots represent the 10 KDM5C mutation cases, the 19 light green dots represent controls, and the two dark green dots represent the benign mutation variants (p.R1546Q). Although the PCA procedure did not use any information on the mutation status, the data distribution shows a clear separation between aberrant mutations and benign mutations and controls. Figure S5: DNA methylation levels at 3 CpG sites in promoter of Long Interspersed Element-1 (LINE-1) as determined by pyrosequencing. The Y-axis is DNA methylation%. Two groups of samples, controls (C; N = 19) and KDM5C mutation cases (K;N = 10) are shown on the X-axis. Figure S6: Regional DNA met [file 1755-8794-6-1-S2.ppt]

## Slide 1
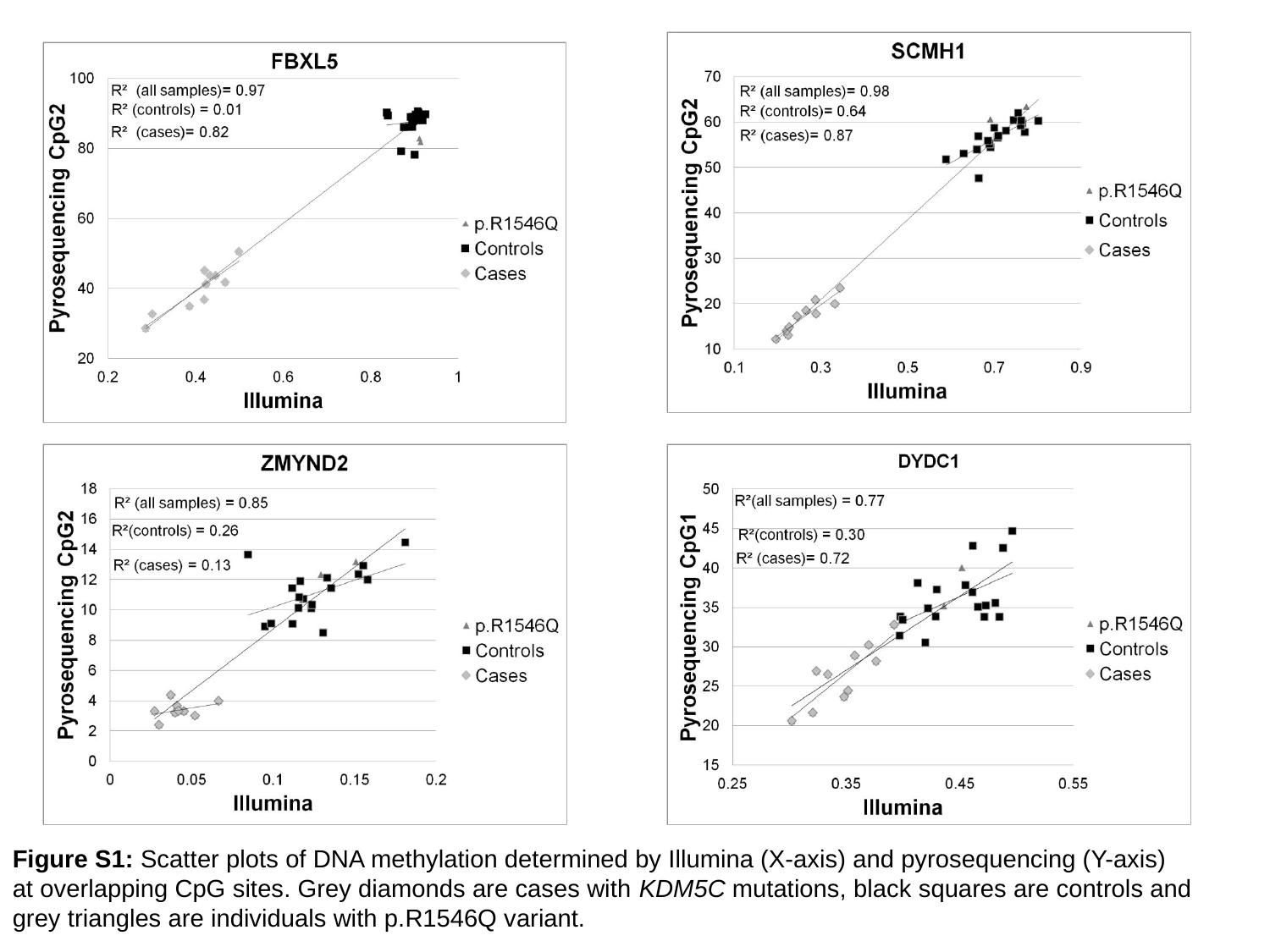

Figure S1: Scatter plots of DNA methylation determined by Illumina (X-axis) and pyrosequencing (Y-axis) at overlapping CpG sites. Grey diamonds are cases with KDM5C mutations, black squares are controls and grey triangles are individuals with p.R1546Q variant.

## Slide 2
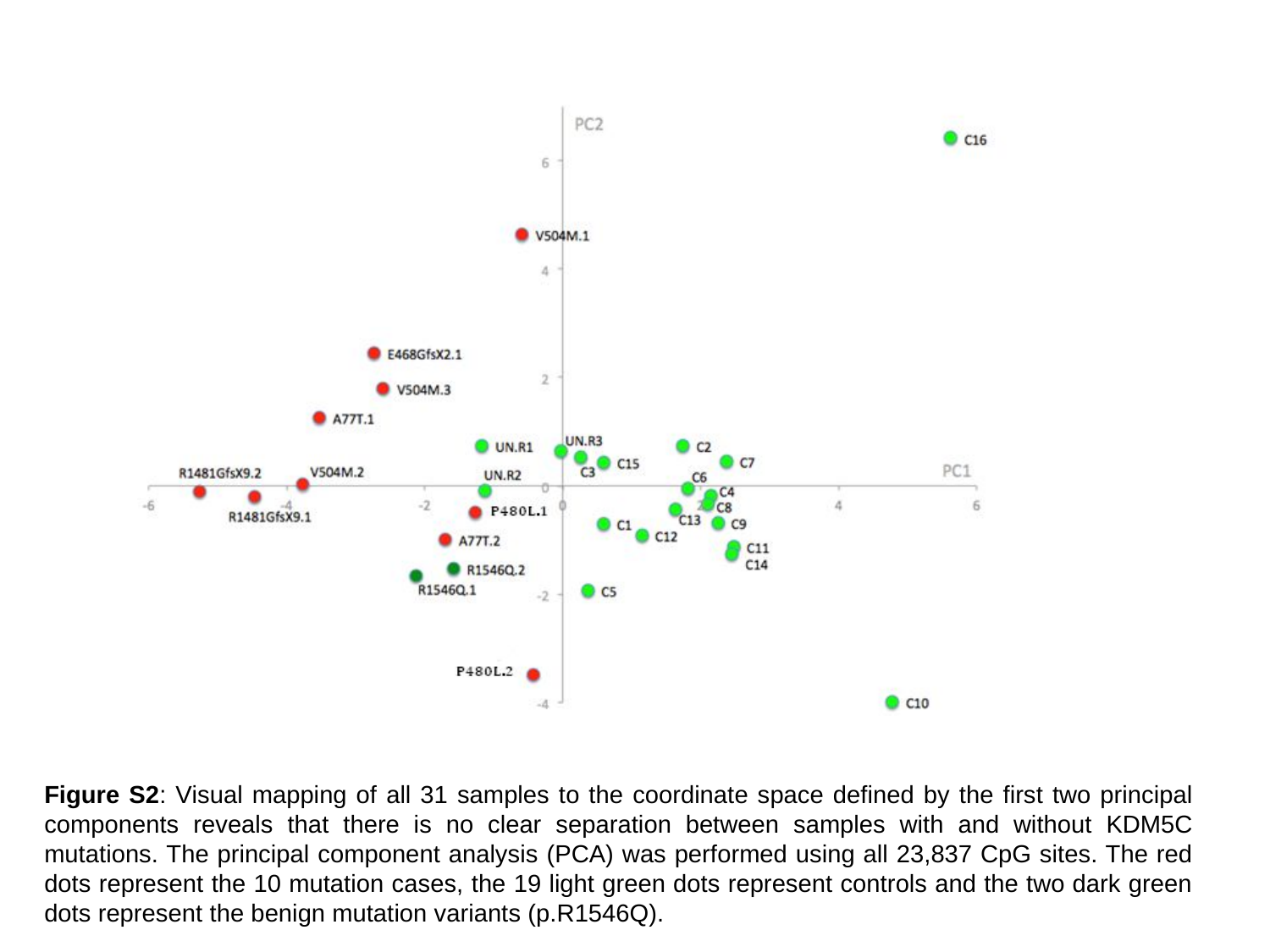

Figure S2: Visual mapping of all 31 samples to the coordinate space defined by the first two principal components reveals that there is no clear separation between samples with and without KDM5C mutations. The principal component analysis (PCA) was performed using all 23,837 CpG sites. The red dots represent the 10 mutation cases, the 19 light green dots represent controls and the two dark green dots represent the benign mutation variants (p.R1546Q).

## Slide 3
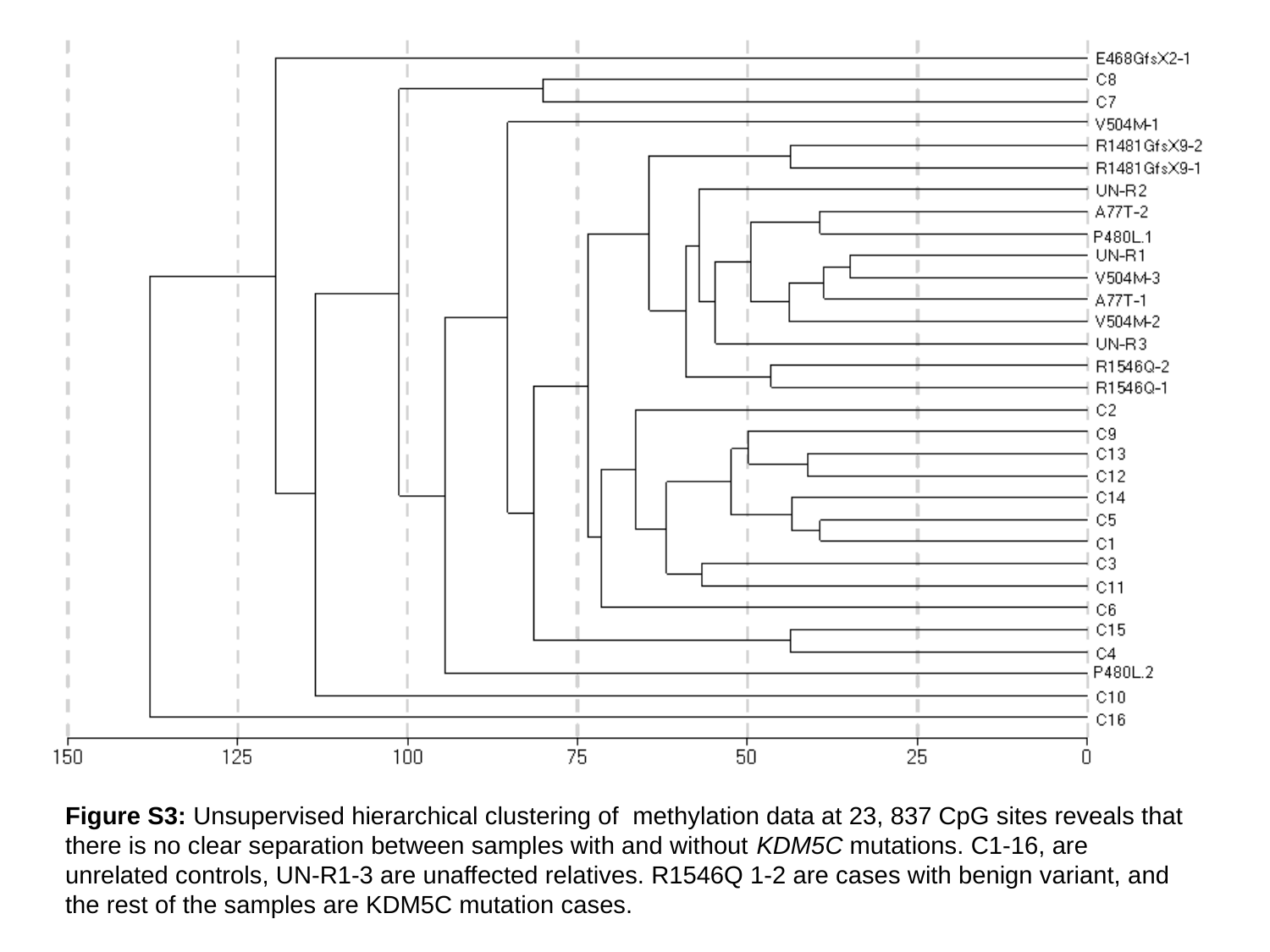

Figure S3: Unsupervised hierarchical clustering of methylation data at 23, 837 CpG sites reveals that there is no clear separation between samples with and without KDM5C mutations. C1-16, are unrelated controls, UN-R1-3 are unaffected relatives. R1546Q 1-2 are cases with benign variant, and the rest of the samples are KDM5C mutation cases.

## Slide 4
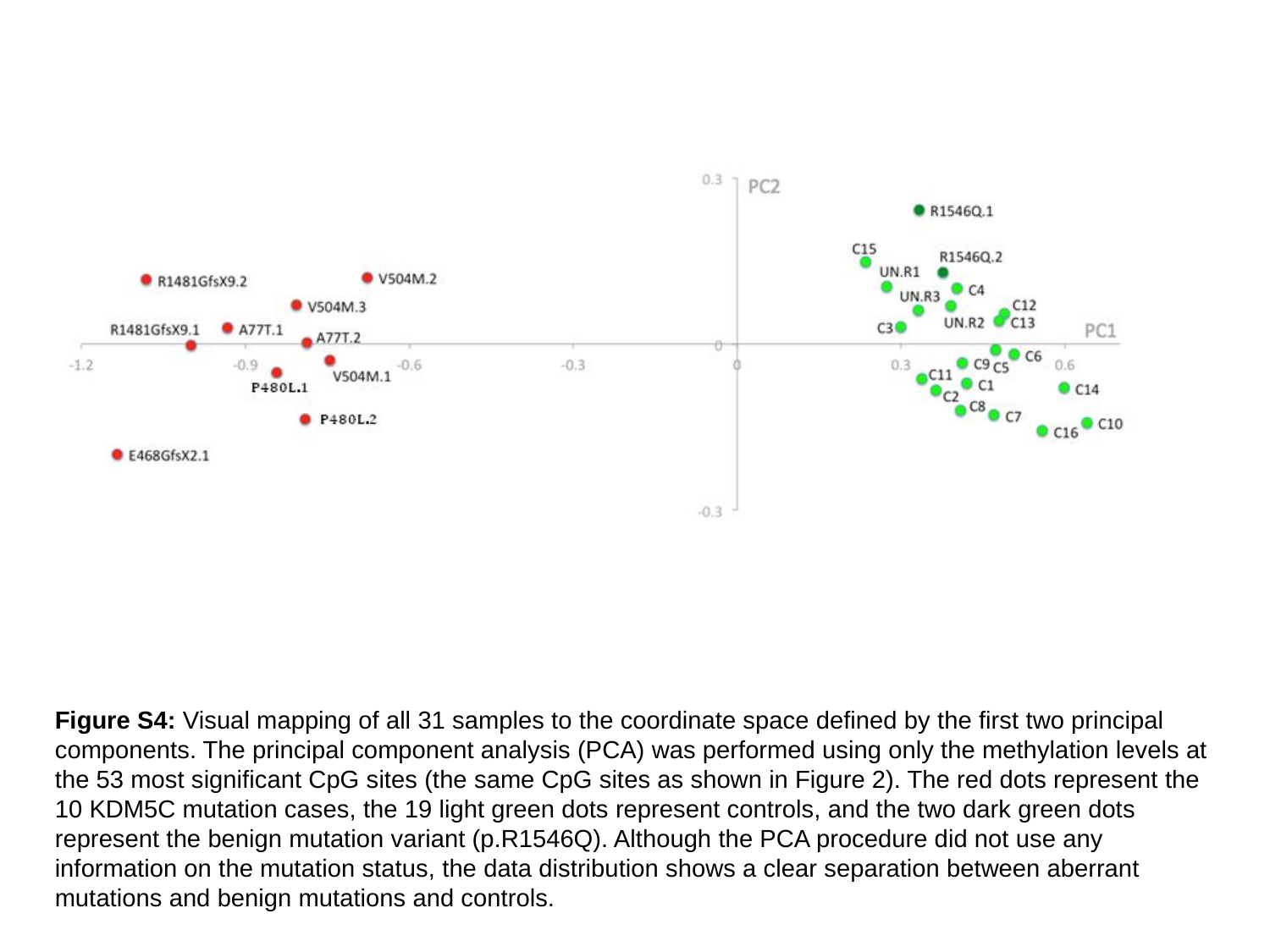

Figure S4: Visual mapping of all 31 samples to the coordinate space defined by the first two principal components. The principal component analysis (PCA) was performed using only the methylation levels at the 53 most significant CpG sites (the same CpG sites as shown in Figure 2). The red dots represent the 10 KDM5C mutation cases, the 19 light green dots represent controls, and the two dark green dots represent the benign mutation variant (p.R1546Q). Although the PCA procedure did not use any information on the mutation status, the data distribution shows a clear separation between aberrant mutations and benign mutations and controls.

## Slide 5
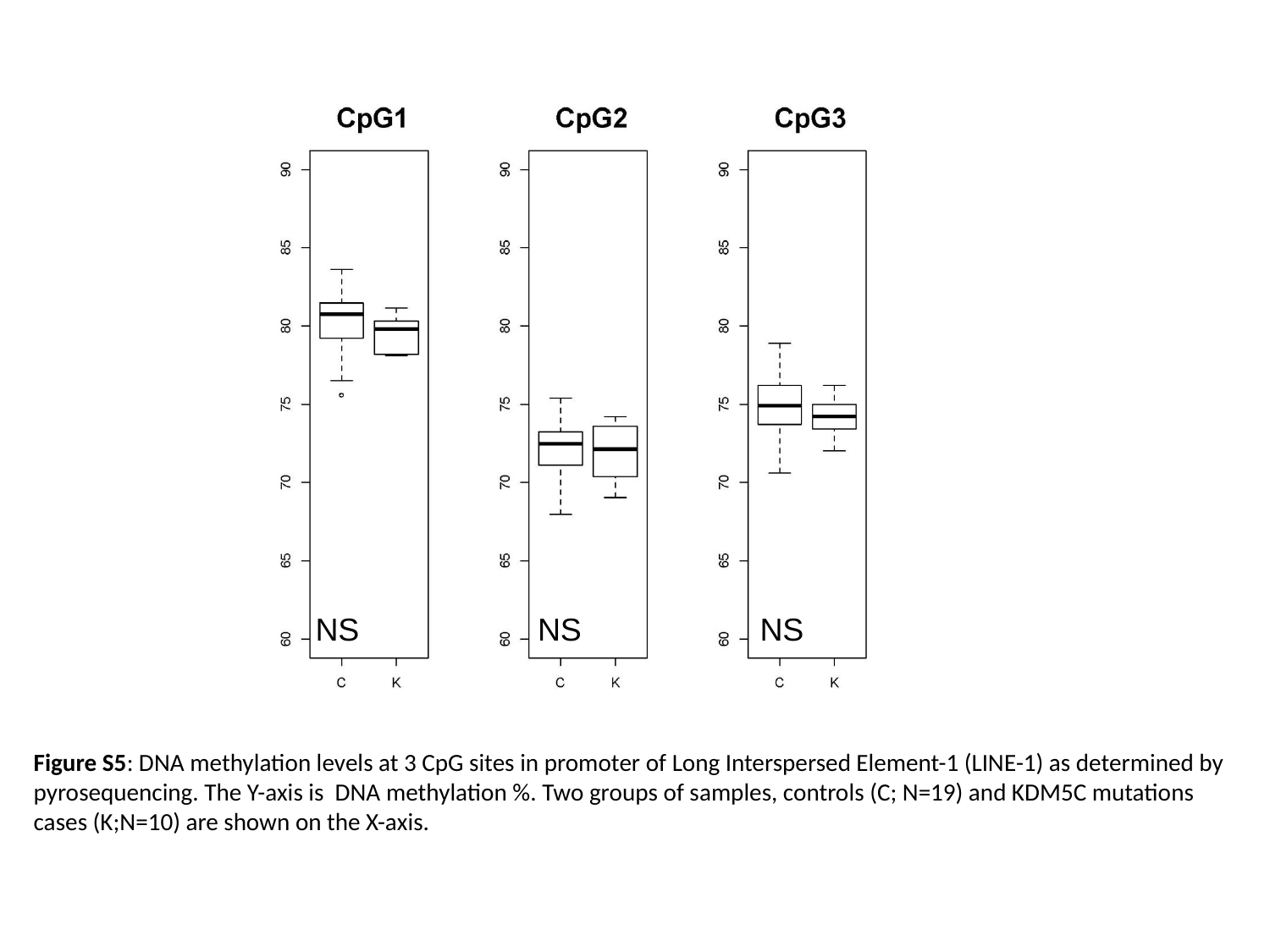

NS
NS
NS
Figure S5: DNA methylation levels at 3 CpG sites in promoter of Long Interspersed Element-1 (LINE-1) as determined by pyrosequencing. The Y-axis is DNA methylation %. Two groups of samples, controls (C; N=19) and KDM5C mutations cases (K;N=10) are shown on the X-axis.

## Slide 6
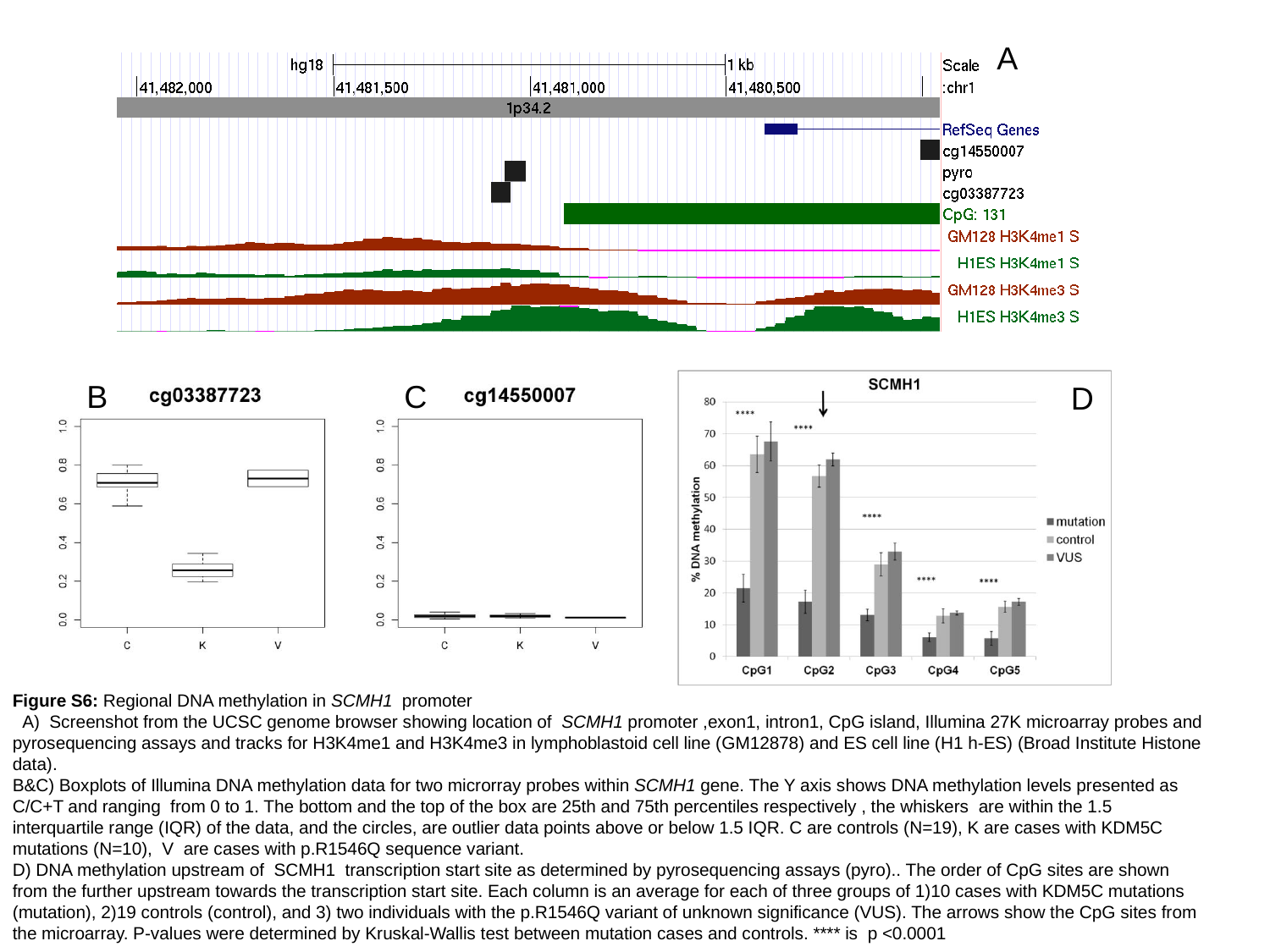

A
B
C
D
Figure S6: Regional DNA methylation in SCMH1 promoter
 A) Screenshot from the UCSC genome browser showing location of SCMH1 promoter ,exon1, intron1, CpG island, Illumina 27K microarray probes and pyrosequencing assays and tracks for H3K4me1 and H3K4me3 in lymphoblastoid cell line (GM12878) and ES cell line (H1 h-ES) (Broad Institute Histone data).
B&C) Boxplots of Illumina DNA methylation data for two microrray probes within SCMH1 gene. The Y axis shows DNA methylation levels presented as C/C+T and ranging from 0 to 1. The bottom and the top of the box are 25th and 75th percentiles respectively , the whiskers are within the 1.5 interquartile range (IQR) of the data, and the circles, are outlier data points above or below 1.5 IQR. C are controls (N=19), K are cases with KDM5C mutations (N=10), V are cases with p.R1546Q sequence variant.
D) DNA methylation upstream of SCMH1 transcription start site as determined by pyrosequencing assays (pyro).. The order of CpG sites are shown from the further upstream towards the transcription start site. Each column is an average for each of three groups of 1)10 cases with KDM5C mutations (mutation), 2)19 controls (control), and 3) two individuals with the p.R1546Q variant of unknown significance (VUS). The arrows show the CpG sites from the microarray. P-values were determined by Kruskal-Wallis test between mutation cases and controls. **** is p <0.0001

## Slide 7
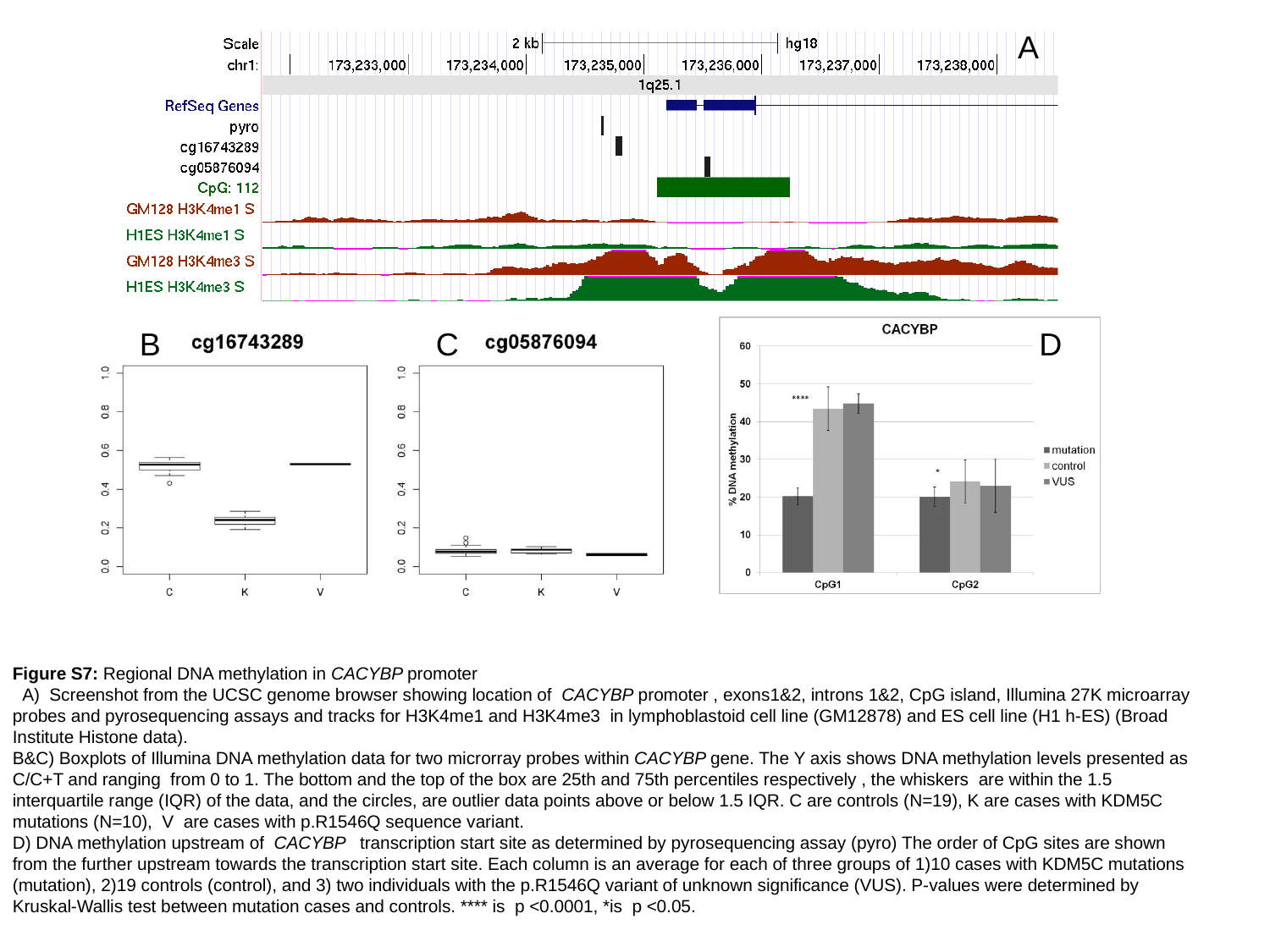

A
B
C
D
Figure S7: Regional DNA methylation in CACYBP promoter
 A) Screenshot from the UCSC genome browser showing location of CACYBP promoter , exons1&2, introns 1&2, CpG island, Illumina 27K microarray probes and pyrosequencing assays and tracks for H3K4me1 and H3K4me3 in lymphoblastoid cell line (GM12878) and ES cell line (H1 h-ES) (Broad Institute Histone data).
B&C) Boxplots of Illumina DNA methylation data for two microrray probes within CACYBP gene. The Y axis shows DNA methylation levels presented as C/C+T and ranging from 0 to 1. The bottom and the top of the box are 25th and 75th percentiles respectively , the whiskers are within the 1.5 interquartile range (IQR) of the data, and the circles, are outlier data points above or below 1.5 IQR. C are controls (N=19), K are cases with KDM5C mutations (N=10), V are cases with p.R1546Q sequence variant.
D) DNA methylation upstream of CACYBP transcription start site as determined by pyrosequencing assay (pyro) The order of CpG sites are shown from the further upstream towards the transcription start site. Each column is an average for each of three groups of 1)10 cases with KDM5C mutations (mutation), 2)19 controls (control), and 3) two individuals with the p.R1546Q variant of unknown significance (VUS). P-values were determined by Kruskal-Wallis test between mutation cases and controls. **** is p <0.0001, *is p <0.05.

## Slide 8
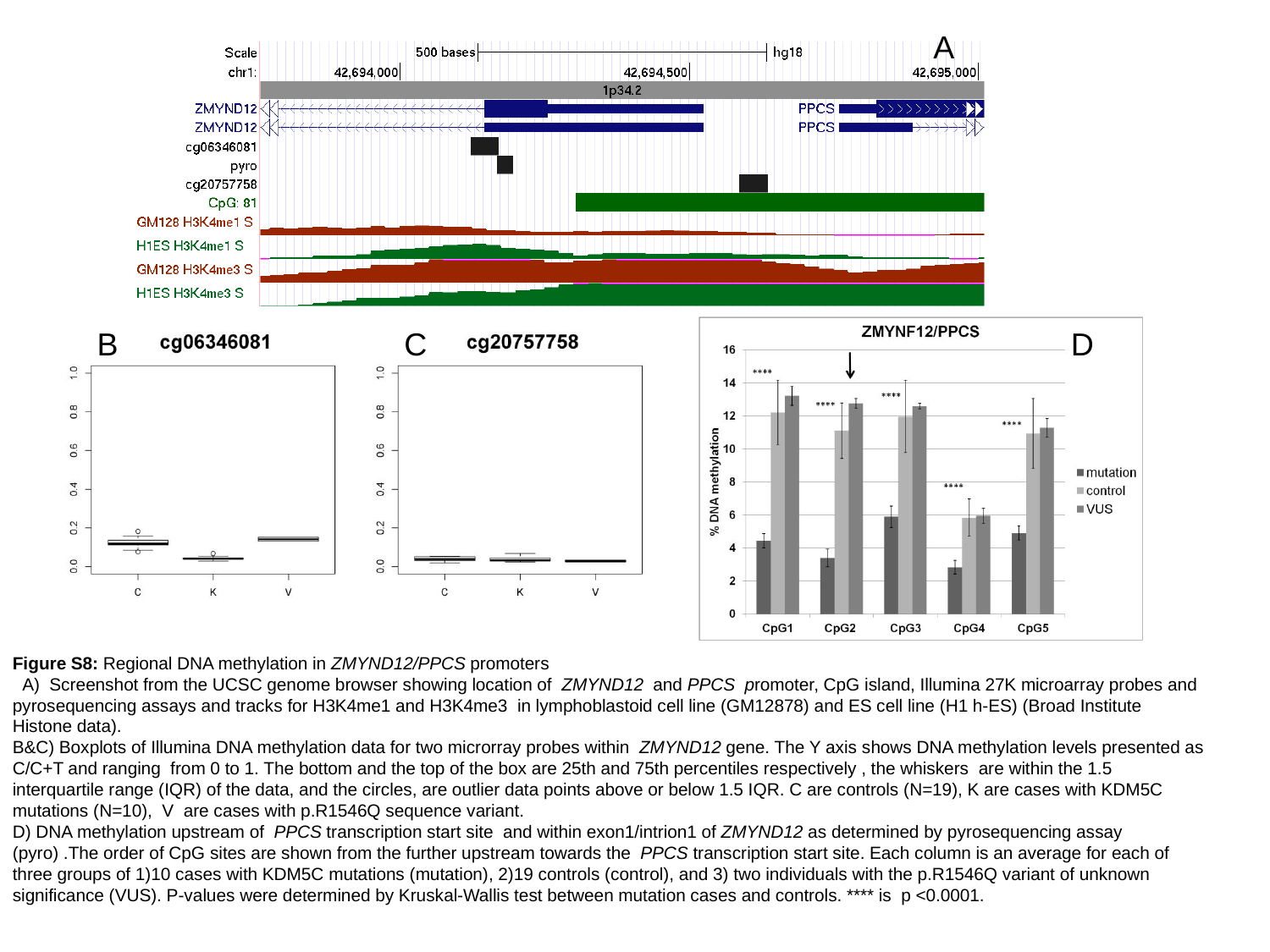

A
B
C
D
Figure S8: Regional DNA methylation in ZMYND12/PPCS promoters
 A) Screenshot from the UCSC genome browser showing location of ZMYND12 and PPCS promoter, CpG island, Illumina 27K microarray probes and pyrosequencing assays and tracks for H3K4me1 and H3K4me3 in lymphoblastoid cell line (GM12878) and ES cell line (H1 h-ES) (Broad Institute Histone data).
B&C) Boxplots of Illumina DNA methylation data for two microrray probes within ZMYND12 gene. The Y axis shows DNA methylation levels presented as C/C+T and ranging from 0 to 1. The bottom and the top of the box are 25th and 75th percentiles respectively , the whiskers are within the 1.5 interquartile range (IQR) of the data, and the circles, are outlier data points above or below 1.5 IQR. C are controls (N=19), K are cases with KDM5C mutations (N=10), V are cases with p.R1546Q sequence variant.
D) DNA methylation upstream of PPCS transcription start site and within exon1/intrion1 of ZMYND12 as determined by pyrosequencing assay (pyro) .The order of CpG sites are shown from the further upstream towards the PPCS transcription start site. Each column is an average for each of three groups of 1)10 cases with KDM5C mutations (mutation), 2)19 controls (control), and 3) two individuals with the p.R1546Q variant of unknown significance (VUS). P-values were determined by Kruskal-Wallis test between mutation cases and controls. **** is p <0.0001.

## Slide 9
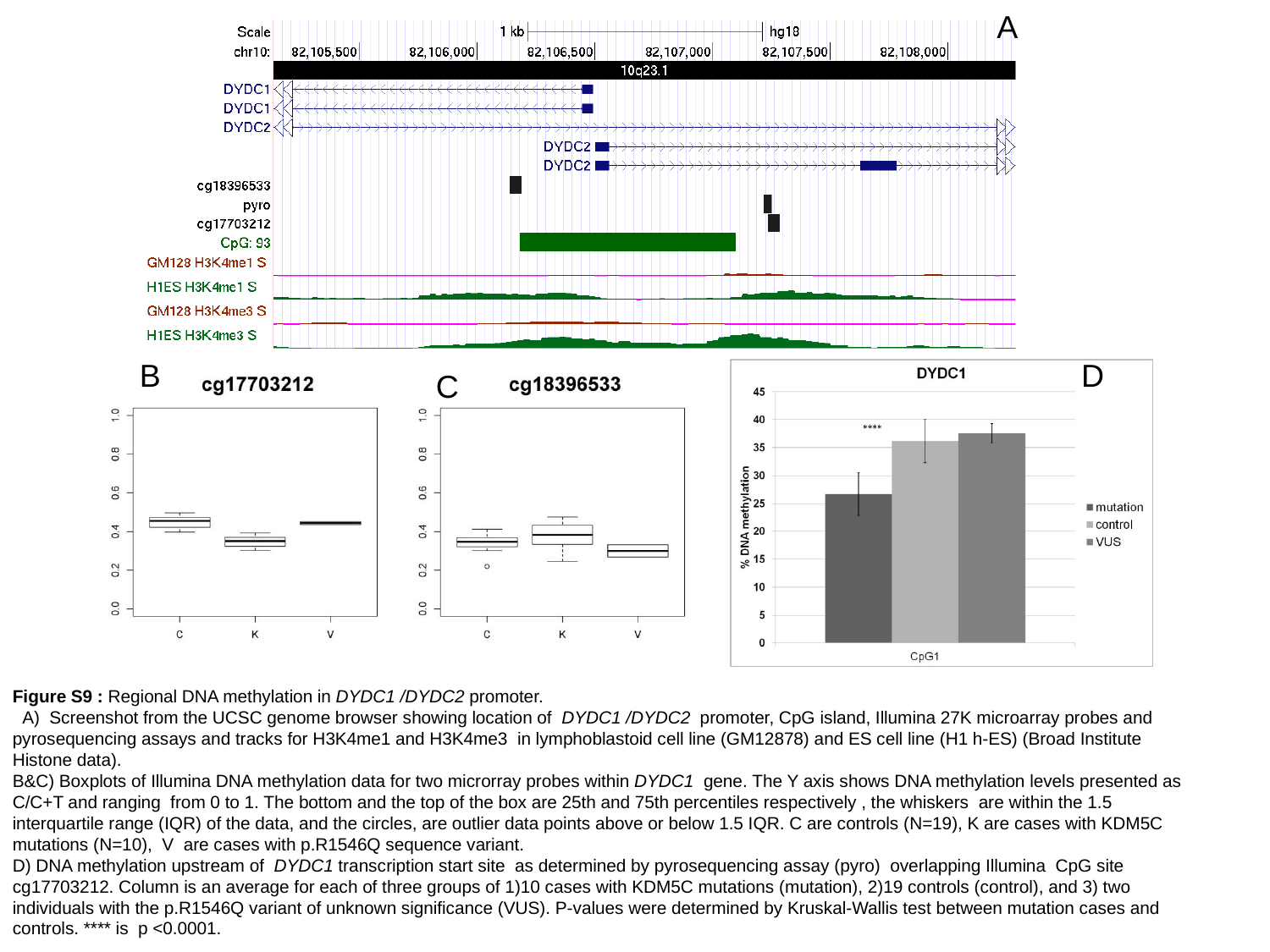

A
B
D
C
Figure S9 : Regional DNA methylation in DYDC1 /DYDC2 promoter.
 A) Screenshot from the UCSC genome browser showing location of DYDC1 /DYDC2 promoter, CpG island, Illumina 27K microarray probes and pyrosequencing assays and tracks for H3K4me1 and H3K4me3 in lymphoblastoid cell line (GM12878) and ES cell line (H1 h-ES) (Broad Institute Histone data).
B&C) Boxplots of Illumina DNA methylation data for two microrray probes within DYDC1 gene. The Y axis shows DNA methylation levels presented as C/C+T and ranging from 0 to 1. The bottom and the top of the box are 25th and 75th percentiles respectively , the whiskers are within the 1.5 interquartile range (IQR) of the data, and the circles, are outlier data points above or below 1.5 IQR. C are controls (N=19), K are cases with KDM5C mutations (N=10), V are cases with p.R1546Q sequence variant.
D) DNA methylation upstream of DYDC1 transcription start site as determined by pyrosequencing assay (pyro) overlapping Illumina CpG site cg17703212. Column is an average for each of three groups of 1)10 cases with KDM5C mutations (mutation), 2)19 controls (control), and 3) two individuals with the p.R1546Q variant of unknown significance (VUS). P-values were determined by Kruskal-Wallis test between mutation cases and controls. **** is p <0.0001.

## Slide 10
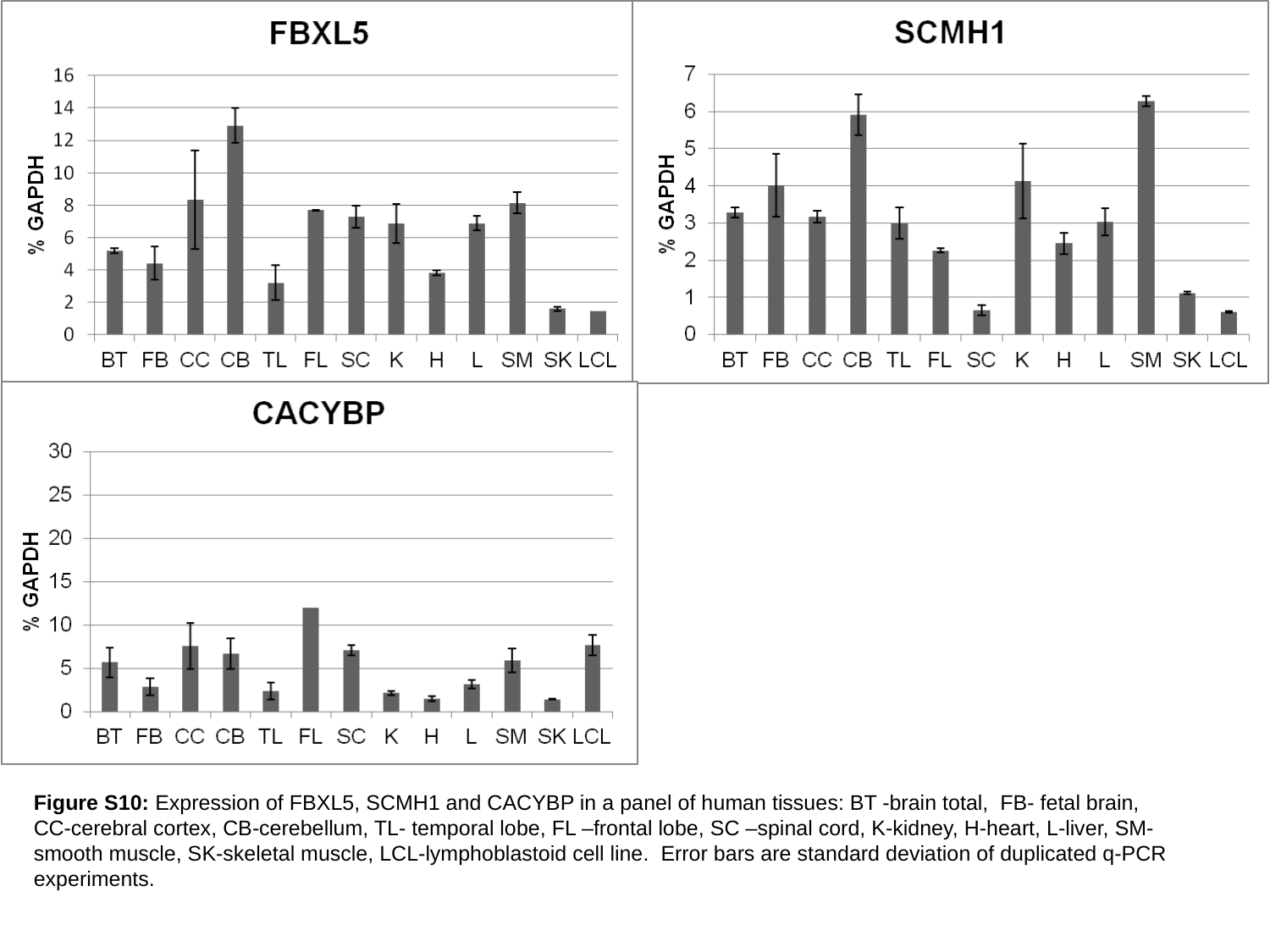

Figure S10: Expression of FBXL5, SCMH1 and CACYBP in a panel of human tissues: BT -brain total, FB- fetal brain, CC-cerebral cortex, CB-cerebellum, TL- temporal lobe, FL –frontal lobe, SC –spinal cord, K-kidney, H-heart, L-liver, SM-smooth muscle, SK-skeletal muscle, LCL-lymphoblastoid cell line. Error bars are standard deviation of duplicated q-PCR experiments.

## Slide 11
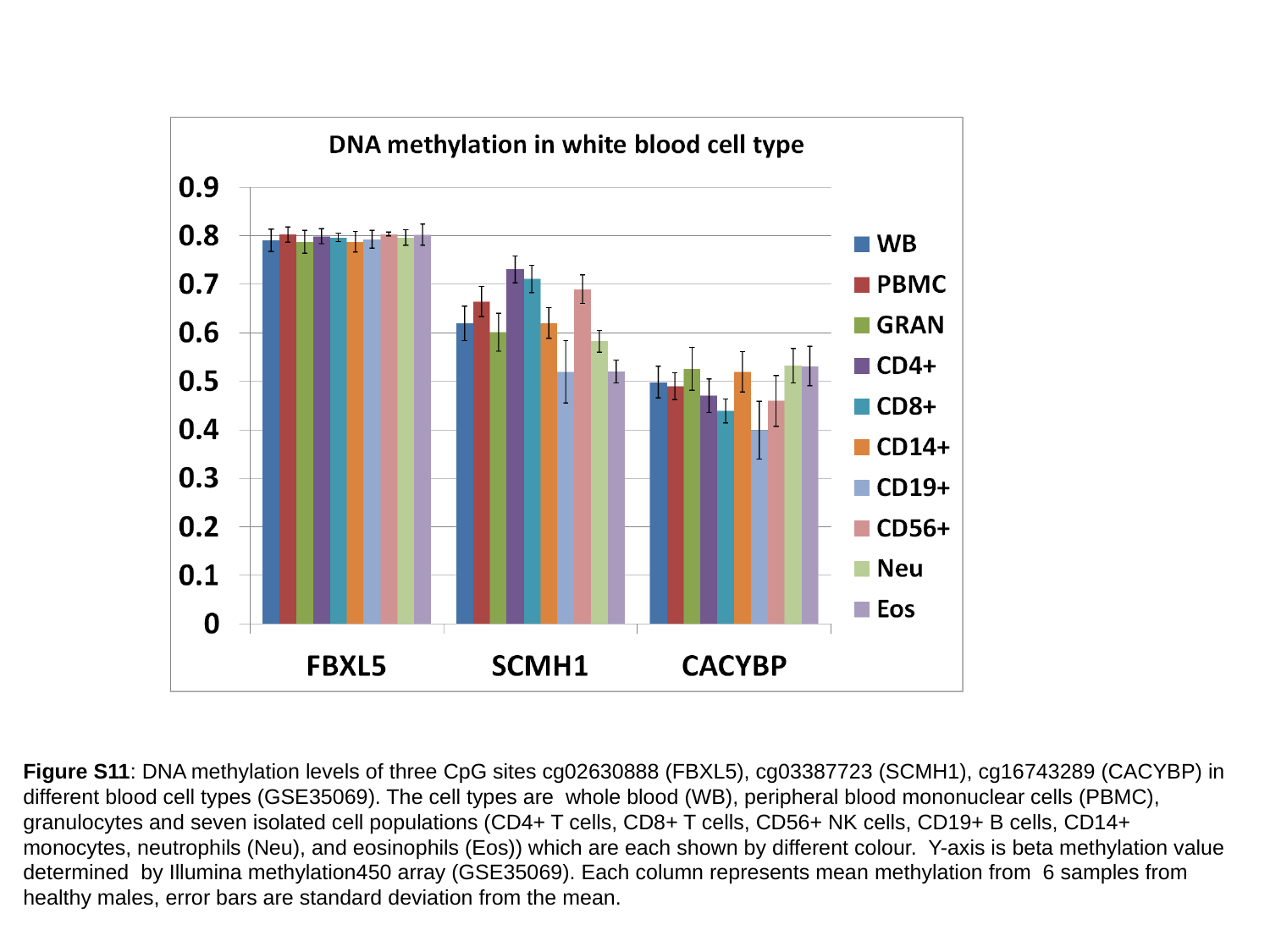

Figure S11: DNA methylation levels of three CpG sites cg02630888 (FBXL5), cg03387723 (SCMH1), cg16743289 (CACYBP) in different blood cell types (GSE35069). The cell types are whole blood (WB), peripheral blood mononuclear cells (PBMC), granulocytes and seven isolated cell populations (CD4+ T cells, CD8+ T cells, CD56+ NK cells, CD19+ B cells, CD14+ monocytes, neutrophils (Neu), and eosinophils (Eos)) which are each shown by different colour. Y-axis is beta methylation value determined by Illumina methylation450 array (GSE35069). Each column represents mean methylation from 6 samples from healthy males, error bars are standard deviation from the mean.
